# Incorporating pollinator movement into connectivity models predicts pollen-mediated gene flow and highlights the importance of regenerating forests in tropical landscapes

**DOI:** 10.1007/s10980-026-02309-y

**Published:** 2026-02-14

**Authors:** Kathryn E. C. Davis, Emil Sloth Thomassen, Helene H. Wagner, Urs G. Kormann, Adam S. Hadley, Matthew G. Betts, Felipe Torres-Vanegas

**Affiliations:** 1 Program in Ecology and Evolution, University of Wyoming, Laramie, WY USA; 2 Department of Ecosystem Science and Management, University of Wyoming, Laramie, WY USA; 3 Department of Biology, Aarhus University, Aarhus C, Denmark; 4 Department of Ecology and Evolutionary Biology, University of Toronto, Mississauga, ON Canada; 5 Applied Ecology Research Group, Swiss Ornithological Institute, Sempach, Switzerland; 6 Biodiversity Section, Department of Natural Resources, Fredericton, NB Canada; 7 Forest Biodiversity Research Network, Department of Forest Ecosystems and Society, Oregon State University, Corvallis, OR USA; 8 Department of Biology, Division of Biodiversity and Evolution, Lund University, Kontaktvägen 10, Lund, Sweden

**Keywords:** Biodiversity conservation, Habitat loss and fragmentation, Landscape genetics, Pollination ecology, Regenerating forest, Species interactions

## Abstract

**Context:**

Pollen-mediated gene flow is crucial for ecological and evolutionary processes and understanding its disruption by anthropogenic disturbances is essential for conservation.

**Objectives:**

In this study, we developed landscape connectivity metrics that incorporated hummingbird movement behaviour to assess how structural (amount and configuration) and functional (species-specific behavioural response) landscape connectivity influence pollen-mediated gene flow in a tropical plant species.

**Methods:**

We adapted the incidence function model (IFM) to develop a set of functional landscape connectivity metrics that integrated field estimates of pollinator movement behaviour. We evaluated whether these metrics outperform structural landscape connectivity metrics for explaining contemporary pollen-mediated gene flow.

**Results:**

The performance of landscape connectivity metrics as predictors of contemporary pollen-mediated gene flow is scale dependent. At the focal patch scale, pollen-mediated gene flow was better predicted by structural connectivity metrics, specifically the area of contiguous mature forest. At the local landscape scale, pollen-mediated gene flow was better predicted by functional connectivity metrics that accounted for hummingbird movement, including gap-crossing probabilities. We found that including regenerating forests and narrow forest elements better explained pollen-mediated gene flow than a focus solely on mature forest.

**Conclusions:**

By integrating hummingbird movement behaviour, we offer a more realistic and nuanced understanding of how landscapes influence pollen-mediated gene flow. We underscore the importance of accounting for pollinator movement behaviour in conservation strategies that aim to preserve pollen-mediated gene flow, and demonstrate that forest regeneration is critical for maintaining functional landscape connectivity in tropical fragmented landscapes.

**Supplementary Information:**

The online version contains supplementary material available at 10.1007/s10980-026-02309-y.

## Introduction

Dispersal is a fundamental life-history process that determines the movement of individuals or propagules, with important short-term ecological and long-term evolutionary consequences for populations (Nathan et al. 2008; Bonte et al. 2012; Auffret et al. 2017). An area of primary interest is to understand the impact of anthropogenic disturbances on the dispersal process, with attention to the role of habitat loss and fragmentation (Damschen et al. 2008; McConkey et al. 2012; Baguette et al. 2013). Together, the effects of habitat loss and fragmentation can produce small and isolated populations at the local and landscape scales (Hadley & Betts 2012; Fahrig 2013). A subsequent reduction in the dispersal capacity among populations can increase random genetic drift, reduce gene flow, and promote inbreeding (Leimu et al. 2010; Schlaepfer et al. 2018; Aguilar et al. 2019), leading to widespread loss of fitness and genetic diversity (Reed & Frankham 2003; Lienert 2004; Lowe et al. 2005; Aguilar et al. 2008). This can render populations less resilient to environmental and demographic stochasticity and increase the likelihood of local extinction (Leimu et al. 2006; Vranckx et al. 2012; Auffret et al. 2017).

The concept of landscape connectivity, which describes the degree to which the landscape facilitates or impedes organism movement (Taylor et al. 1993), is fundamental to understand the impact of habitat loss and fragmentation on the dispersal process (Auffret et al. 2017). Structural landscape connectivity refers to the amount and spatial configuration of distinct habitat areas within a landscape (Taylor et al. 1993), which often influence ecological and life-history processes that are critical for organism movement (Bélisle 2005; Nathan et al. 2008; Vasudev et al. 2015). Given this influence, potential functional landscape connectivity incorporates species-specific life-history information to more accurately describe the dispersal process among habitat areas (Taylor et al. 1993; Tischendorf & Fahrig 2000). Potential functional landscape connectivity is thus dependent on both the structure of the landscape (*i.e.,* amount and spatial configuration) and how it alters the movement behaviour of the species under consideration, as it models the species-specific behavioural response (Calabrese & Fagan 2004; Bélisle 2005; Auffret et al. 2017). Actual functional landscape connectivity reflects the realized movement of individuals and their genes across the landscape (Calabrese & Fagan 2004), which is influenced by individual behaviour and species interactions (Auffret et al. 2017). Thus, to comprehensively understand the impact of habitat loss and fragmentation on the dispersal process, it is important to integrate characteristics of individual behaviour with the concept of landscape connectivity (Hadley & Betts 2012; Auffret et al. 2017).

For most plant species, the dispersal process involves two separate life-history processes that often require the participation of biotic vectors (Ollerton et al. 2011; Tong et al. 2023): (1) pollen transport (*i.e.,* the pollination process); and (2) seed dispersal. Thus, the dispersal process in most plant species depends on the structural components of landscape connectivity as well as the potential functional components of the focal species, including interactions with biotic vectors and their natural history (Hadley & Betts 2012; Auffret et al. 2017; Minnaar et al. 2019). Pollen transport and seed dispersal can have independent responses to the structural components of landscape connectivity, as they are mediated by distinct biotic vectors (Ennos 1994; Ghazoul 2005; Krauss et al. 2009; Markl et al. 2012; Auffret et al. 2017). That is, the biotic vectors that mediate pollen transport and seed dispersal respond to habitat loss and fragmentation in unique and species-specific ways, often operating at distinct spatial scales determined by their movement behaviour (Ennos 1994; Aguilar et al. 2006; Schupp et al. 2010; Hadley & Betts 2012; Browne et al. 2018). Indeed, previous work has shown that the relative importance of the structural and functional components of landscape connectivity in explaining biodiversity patterns in fragmented landscapes is scale dependent (Mühlner et al. 2012).

Attention to the impact of landscape connectivity on pollen transport is particularly important, as this process is an essential constituent of the biodiversity of terrestrial ecosystems (Kremen et al. 2007; Mitchell et al. 2009; Karron et al. 2012). For most plant species, pollen transport typically occurs across a greater distance compared to seed dispersal (Ennos 1994; Bittencourt & Sebbenn 2007; Hanson et al. 2008; Browne et al. 2018; Nakanishi et al. 2021) and is thought to play a more significant role in the movement of genes across the landscape (Sork & Smouse 2006; Hamrick 2010; Sork et al. 2015; Sujii et al. 2021). Thus, it is important to isolate the role of pollen transport and seed dispersal as distinct life-history processes that maintain landscape connectivity for most plant species. This is particularly important for tropical plant species, which are underrepresented in the literature and highly susceptible to anthropogenic disturbances (Aguilar et al. 2006; Winfree et al. 2011; Hadley & Betts 2012; Teixido et al. 2022).

Most tropical landscapes today consist of a mosaic of landcover types that often include anthropogenic disturbances (*e.g.,* agriculture, pastures, settlements) embedded within the remnants of mature forests (*e.g.,* considered natural habitat areas) and regenerating forests (*e.g.,* recovering following past disturbance) (Asner et al. 2009; Zahawi et al. 2015). Although mature forests often provide optimal habitat for many pollinator species (Bawa 1990; Hadley et al. 2018; Huh et al. 2023; Ulyshen et al. 2023), the extent to which regenerating forests facilitate pollen transport and pollen-mediated gene flow across tropical landscapes remains poorly understood. Thus, a comprehensive assessment of landscape connectivity in tropical plant species is contingent on understanding how distinct landcover types, particularly mature and regenerating forests, contribute to the maintenance of pollen-mediated gene flow (Kormann et al. 2016; Mayhew et al. 2019; Rosenfield et al. 2023). This will provide insights to ensure the persistence of ecological and evolutionary processes in fragmented tropical landscapes.

Here, we evaluated the impact of the structural and functional components of landscape connectivity on contemporary pollen-mediated gene flow in *Heliconia tortuosa* Griggs (Heliconiaceae), a hummingbird-pollinated tropical plant. We integrated data on pollinator movement behaviour into functional landscape connectivity metrics and evaluated whether these outperform structural landscape connectivity metrics at explaining contemporary pollen-mediated gene flow in *H. tortuosa*. We adapted the incidence function model (IFM) (Hanski 1994), which quantifies the connectivity of a focal habitat patch with its neighbours based on their size and the distances separating them, to develop a set of structural and functional landscape connectivity metrics for our study system, conceptually aligning with a neighbourhood-based landscape genetics approach (Wagner & Fortin 2013). We used these metrics to quantify landscape connectivity at two spatial scales: (1) the focal habitat patch (*i.e.,* intra-patch connectivity); and (2) the local landscape (within 1 km, representing the maximum daily movement range of hummingbirds) (Volpe et al. 2016).

First, we applied the IFM to define landscape connectivity metrics that integrate the spatial configuration of trapliner habitat (*i.e.,* habitat used by hummingbird pollinator species that are both habitat specialists and exhibit traplining foraging behaviour) with data on pollinator movement behaviour (Leimberger 2022). Specifically, we considered three aspects of movement behaviour (*i.e.,* daily flight distance, home range length, and gap-crossing probability) and two alternative definitions of trapliner habitat. With the latter, we aimed to assess whether regenerating forests and narrow forest elements, which are known to facilitate pollinator movement in this study system (Volpe et al. 2014; Kormann et al 2016), are important for maintaining plant functional connectivity, or if only mature forests matter. Second, we used previously published genetic data (Torres‐Vanegas et al. 2021) to quantify contemporary pollen-mediated gene flow (*i.e.,* actual functional landscape connectivity). Third, we compared to what degree these gene flow patterns can be explained by alternative metrics of structural and potential functional connectivity. This landscape genetic approach to connectivity analysis can provide insights to identify the structural and functional components of landscape connectivity that are most relevant to protect pollen transport in *H. tortuosa* and other animal-pollinated plants. In an era of prevalent habitat loss and fragmentation, it is essential to understand the impact of landscape connectivity on contemporary pollen-mediated gene flow and leverage these insights to protect the long-term viability of plant populations (Leimu et al. 2010; Auffret et al. 2017; Moreno-Mateos et al. 2020).

## Methods

### Study area

This study was conducted in an area surrounding the Organization for Tropical Studies Las Cruces Biological Station, in southern Costa Rica (8°47′7″ N, 82°57′32″ W). The study area (approx. 31,000 ha) was originally covered by Pacific premontane neotropical forest. The non-forested matrix is dominated by pasture and agriculture, and underwent dramatic deforestation that primarily occurred from 1960 to 1980 (Hadley et al. 2014; Zahawi et al. 2015). Today, only 20% of the original old-growth forest (*i.e.,* mature forest cover present before 1947) remains in the study area (Zahawi et al. 2015). Since 1980, deforestation has been partially offset through secondary forest growth (*i.e.,* regenerating forest), which today represents 30% of the remaining habitat (Zahawi et al. 2015; Reid et al. 2019).

### Study system

*Heliconia tortuosa* is a perennial and hermaphroditic herb exclusively found in the understory of premontane neotropical forests, where it occurs individually or in clonal clumps (Stiles 1975). Across the study area, *H. tortuosa* is one of the most common hummingbird-pollinated plants (Leimberger et al. 2023). During peak flowering season (February to May), individuals typically produce one or two inflorescences. Each inflorescence holds up to 12 bracts, each subtending up to 15 flowers that are fertile for a single day (Stiles 1975). Upon successful pollination, *H. tortuosa* produces fleshy fruits with up to three seeds, and seed dispersal is mediated by several generalist frugivore birds (Arias Medellín, 2022).

*Heliconia tortuosa* can reproduce clonally and is partially self-compatible (Kress 1983). Self-fertilization is possible between different flowers of the same individual (geitonogamy), yet pollinator exclusion experiments have shown an absence of self-fertilization within the same flower (autogamy) (Kress 1983; Betts et al. 2015). Hand pollination often does not lead to ovule fertilization, unless combined with a hummingbird visit, which suggests that hummingbirds are required for successful pollination (Betts et al. 2015). This tropical forest herb is pollinated by two hummingbird functional groups (sensu Fenster et al. 2004) that differ in their morphology and movement behaviour (Betts et al. 2015; Leimberger et al. 2023). Territorial hummingbirds (*e.g., Amazilia tzacatl*, *Heliodoxa jacula*, *Phaeochroa cuvierii* and *Thalurania colombica*) are considered habitat generalists (*i.e.,* associated with both mature and regenerating forest) that aggressively defend small areas (< 100 m in diameter) containing high floral resource density (Betts et al. 2015; Jones et al. 2022; Leimberger et al. 2023). Traplining hummingbirds (*e.g., Campylopterus hemileucurus, Phaethornis guy* and *Phaethornis longirostris*) are mostly considered habitat specialists that are associated with mature forest (Morrison & Mendenhall 2020; Jones et al. 2022). Traplining hummingbirds typically forage across long-distance routes (up to 1 km per day) to acquire nectar (Volpe et al. 2014; Betts et al. 2015) and are thought to engage in repeated sequential visits to resource locations (Torres-Vanegas et al. 2023). In *H. tortuosa*, traplining hummingbirds account for approx. 85% of flower visits (Leimberger et al. 2023) and are characterized by large body size (*e.g.,* 11–15 cm) and specialized bill morphology (*i.e.,* long and curved) (Betts et al. 2015; Hadley et al. 2018).

### Study design

Our study design was based on previous work in the study area. Hadley et al., (2014) used a stratified-random sampling design to select 40 focal patches classified as mature forest. We used a subset of 30 focal patches chosen for long-term research in the study system (Figs. S1, S2). These ranged from 0.6 ha to > 1,300 ha in size and the percentage of forest cover within a 1 km radius ranged from 7.8% to 74.4% (Figs. S1, S2).

During the 2013 flowering season, we sampled 25 focal patches. During the 2016 flowering season, we resampled 13 focal patches and included five additional focal patches, which resulted in a total of 30 focal patches. In each patch, we identified a road access point from which we randomly selected a location (hereafter ‘sampling site’) anywhere within a distance of up to 500 m (Hadley et al. 2014). From each sampling site, we marked the nearest five flowering *H. tortuosa* individuals (hereafter ‘maternal plants’). To avoid clonal individuals, we required a minimum distance of 1 m among the selected maternal plants. At the end of each flowering season, we sampled leaf tissue from each maternal plant and covered a single inflorescence to avoid fruit removal. Once fruits were mature, we randomly selected two bracts per inflorescence and collected the seeds from all fruits. In 2013, we sampled 87 maternal plants, while 71 new maternal plants were sampled in 2016. We selected an average of 10 seeds per maternal plant (range 5 to 21) for DNA extraction and genotyping, resulting in a total of 1,584 seeds (2013: 770 seeds; 2016: 814 seeds).

### Genetic data

In this study, we analyze the genetic data included in Torres‐Vanegas et al., (2021), which evaluated the impact of structural landscape connectivity on contemporary pollen-mediated gene flow in *H. tortuosa*. We expand on this by estimating potential functional landscape connectivity metrics that incorporate data on hummingbird movement behaviour and compare how well these metrics predict contemporary pollen-mediated gene flow (*i.e.,* actual functional landscape connectivity) in *H. tortuosa*.

Genomic DNA was extracted from all sampled maternal plants and selected seeds (embryos dissected) using the QIAGEN DNeasy Plant Mini Kit (QIAGEN). These materials were genotyped at 11 microsatellite loci following Torres-Vanegas et al., (2019). We obtained pollen haplotypes by subtracting the genetic contribution of each maternal plant from the multilocus genotype of each corresponding seed, using the minus.mom function of the gstudio package (Dyer 2016) in R 4.3.3. (R Core Team 2023). Based on the allele frequencies of multilocus pollen haplotypes, we estimated the haplotype diversity (*h*) of pollen pools sampled by each maternal plant (Torres‐Vanegas et al. 2021). This measure corresponds to the probability (averaged across all loci) that the paternal alleles of two randomly chosen seeds from the same maternal plant are different (Nei 1987). We used MLTR 3.4 (Ritland 2002) to estimate biparental inbreeding (*t*_*m*_—*t*_*s*_) for the seeds sampled from each maternal plant (Torres‐Vanegas et al. 2021), which corresponds to a measure of the frequency of mating among related individuals.

We combined the pollen pools sampled by the maternal plants at each sampling site to estimate contemporary pollen-mediated gene flow at the sampling site level. For sampling sites that were considered in 2013 and 2016, we combined the pollen pools sampled by the maternal plants across the years. Note that the maternal plants considered in 2013 and 2016 corresponded to distinct plant individuals (Torres‐Vanegas et al. 2021).

### The composition of the pollinator community

Based on hummingbird capture data from Hadley et al., (2018), we described the species composition of the pollinator community within a subset of 13 focal patches, which were sampled from February to March in 2010 and 2011. In each focal forest patch, ten mist nets were placed within three meters of hummingbird-visited flowers and each captured hummingbird was identified to species level (for details see Hadley et al. 2018). These were classified as low- or high-mobility, according to their median daily foraging distance (Betts et al. 2015; Torres‐Vanegas et al. 2021). Species with a median daily foraging distance > 0.5 km were classified as high-mobility (*Campylopterus hemileucurus, Phaethornis guy*, and *Phaethornis longirostris;* hereafter referred to as 'trapliners'), while all other species (*Amazilia decora*, *Amazilia edward*, *Amazilia tzacatl*, *Heliodoxa jacula*, *Phaeochroa cuvierii,* and *Phaethornis striigularis; *where all but the last are 'territorial') were classified as low-mobility (Betts et al. 2015; Torres‐Vanegas et al. 2021). To represent the species composition of the pollinator community, we used the number of hummingbird captures to estimate the proportion of high-mobility hummingbirds at each focal patch, which included a small-sample correction that involved adding four pseudo-observations (Torres‐Vanegas et al. 2021). In addition, the maximum daily movement range of traplining hummingbirds (1 km) (Volpe et al. 2016) was used to parameterize the landscape connectivity metrics, reflecting an upper bound of daily movement for the most common and effective pollinators of *H. tortuosa*, *P. guy* and *C. hemileucurus* (Betts et al. 2015; Leimberger et al. 2023).

### Landscape data

We used digitized landcover types from Hadley et al., (2018) that were based on historical and current forest cover in the study area over a sixty-seven year period (Zahawi et al. 2015). This historical characterization was based on aerial imagery from five time periods (Zahawi et al. 2015): (1) 1947; (2) 1960; (3) 1980; (4) 1997; and (5) 2014, derived from high-resolution Google Earth images. While the datasets were collected in different years, the rate of landcover change in the study area has slowed in recent decades and shifted from forest loss to regeneration (Zahawi et al. 2015). Thus, any changes in forest cover between sampling and landcover mapping are minor and unlikely to have influenced the results.

The digitized landcover types included: (1) mature forest (*i.e.,* continuous forest cover older than 1980 that remains in the study area); (2) regenerating forest (*i.e.,* forest cover growth since 1980); (3) narrow forest elements (*i.e.,* living fencerows and riparian strips); (4) arable land (*i.e.,* primarily coffee and banana); and (5) pasture (*i.e.,* areas grazed by cattle). The narrow forest elements were digitized based on a combination of historical forest cover and high-resolution Google Earth images (Hadley et al. 2018). We used the landcover types to define habitat for traplining hummingbirds in two ways. Given that the relative availability of high-mobility hummingbirds is greater in mature forests compared to regenerating forests (Jones et al. 2022), we considered a ‘narrow’ definition of trapliner habitat that solely included mature forest. However, traplining hummingbirds can also utilize other landcover types to sustain their daily movement patterns across the landscape (Hadley & Betts 2009; Volpe et al. 2014; Kormann et al. 2016). Thus, we also considered a ‘broad’ definition of trapliner habitat that included mature forest, regenerating forest, and narrow forest elements. Landcover types classified as agriculture and pasture were always excluded from the trapliner habitat definition.

We defined a ‘local landscape’ that surrounded each sampling site as the area within a 1 km radius of the median GPS coordinate of the sampled maternal plants. This distance corresponds approx. to the maximum daily movement range of traplining hummingbirds, as determined by radio-telemetry studies (Hadley & Betts 2009; Betts et al. 2015; Volpe et al. 2016), and is assumed to contain the pollen donors that are available (*i.e.,* functionally connected) to the sampled maternal plants. The estimation of landscape connectivity metrics was restricted to the local landscape that surrounded each sampling site (except total patch size). In this study, the digitized landcover types in the local landscapes were rasterized with a 10 m resolution (*i.e.,* raster cells of 10 m by 10 m) and separately classified according to the ‘narrow’ and ‘broad’ definitions of trapliner habitat, so that each pixel was classified as either 1 for trapliner habitat or 0 for trapliner non-habitat (Leimberger 2022).

### Landscape connectivity metrics

**Fig. 1:**
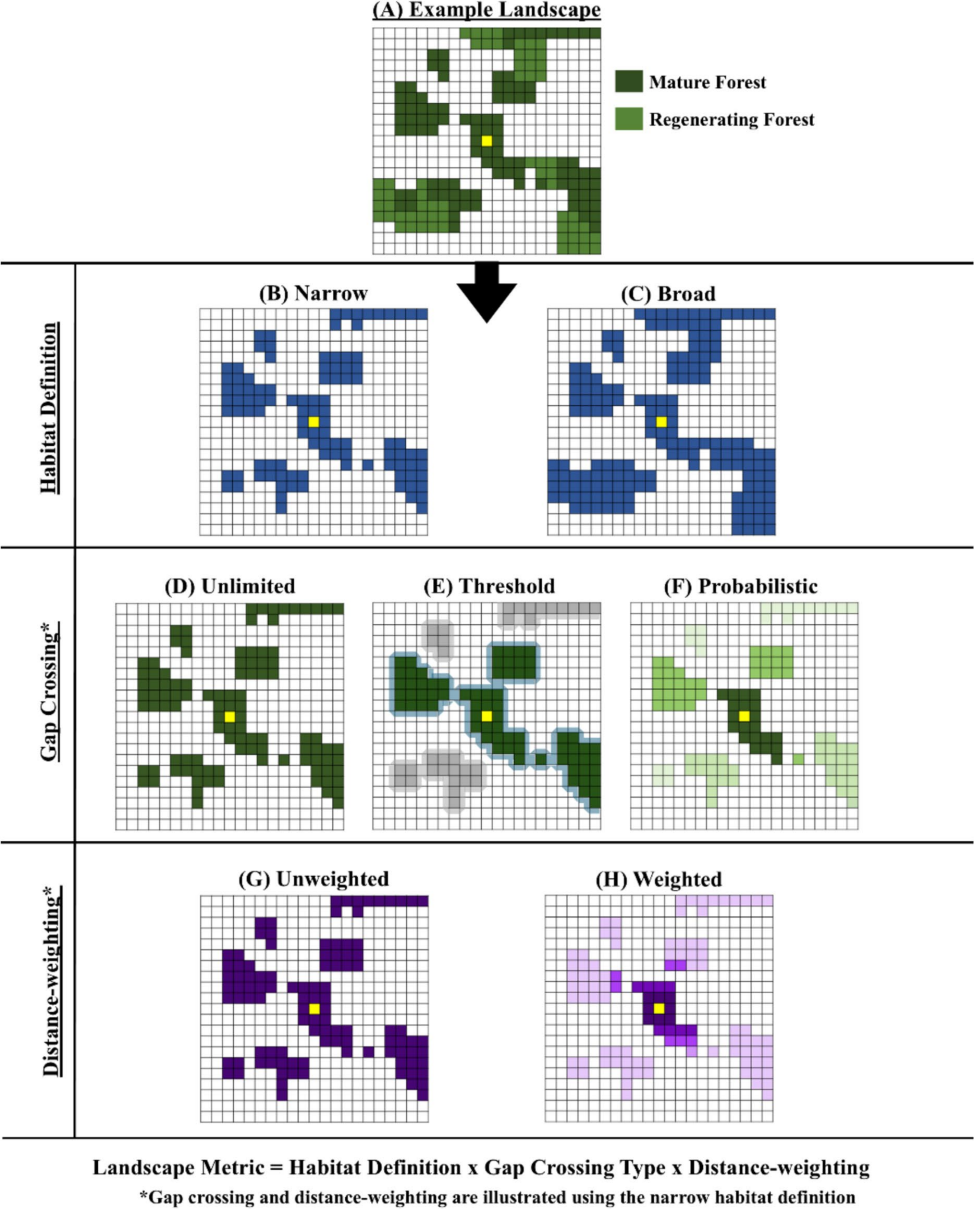
A conceptual representation of the factorial design of the local landscape connectivity metrics considered in this study. Each square panel shows a rasterized landscape with potential habitat pixels (colored) and non-habitat pixels (white); the yellow pixel indicates the sampling site within the focal patch. **A** From a rasterized local landscape containing both mature and regenerating forest, we first applied either a **B** ‘narrow’ definition of trapliner habitat, corresponding to mature forest, or a **C** ‘broad’ definition, corresponding to all forest types. Then, one of three gap-crossing approaches was applied to the map of habitat patches (illustrated in this Figure with the ‘narrow’ habitat definition): **D** unlimited gap-crossing, wherein hummingbirds move freely between patches regardless of the gaps between them; **E** a gap-crossing threshold, wherein each habitat patch is buffered by 25 m (shown approx.) and patches are only considered connected if their buffers overlap (shown in light blue); and **F** a probabilistic gap-crossing approach, wherein patches separated by smaller gaps contribute more (dark green) to landscape connectivity than patches separated by larger or multiple gaps (light green). Finally, to the combination of habitat definition and gap-crossing approach, we either: **G** weighted all habitat pixels equally; or **H** applied a distance-based kernel, wherein pixels located closer to the sampling location within the focal patch contributed more (dark purple) to landscape connectivity than pixels located farther away (light purple). Thus, we ultimately had 12 local landscape connectivity metrics that varied by habitat definition, gap-crossing approach, and distance-weighting

A broad range of landscape connectivity metrics have been proposed (Calabrese & Fagan 2004; Kindlmann & Burel 2008; Keeley et al. 2021), representing structural and potential functional landscape connectivity. Previous work in the study area has evaluated the impact of two landscape connectivity metrics on contemporary pollen-mediated gene flow in *H. tortuosa* (Torres‐Vanegas et al. 2021): (1) patch size in ha (log-transformed), a purely structural landscape connectivity metric; and (2) the amount of forest cover within a 1 km radius, a simple functional landscape connectivity metric incorporating the maximum daily movement range of traplining hummingbirds in the study area (Hadley et al. 2014; Volpe et al. 2016). In this study, we conceptualize and quantify functional landscape connectivity metrics that integrate the amount and spatial configuration of trapliner habitat with their movement behaviour (Fig. 1; Table 1). Thus, we capitalize on the abundant data available for our study system to improve our understanding of the impact of habitat loss and fragmentation on contemporary pollen-mediated gene flow as an important aspect of plant functional connectivity.

**Table 1:** Summary of all landscape connectivity metrics, where each quantifies the amount (ha) of trapliner habitat that is assumed to be functionally connected to a sampling site. We quantified three intra-patch connectivity metrics (top) and 12 local landscape connectivity metrics (bottom). Intra-patch connectivity metrics are sorted from purely structural to the most complex functional metrics, *i.e.,* those with the most detailed assumptions about pollinator movement behaviour. Local landscape metrics are listed according to gap-crossing assumptions (three levels), which were combined with trapliner habitat definition (‘narrow’ vs ‘broad’) and distance-weighting (without/with) in a full factorial design. The combination of probabilistic gap-crossing with a ‘broad’ trapliner habitat definition and with distance-weighting represents the most complex functional metric

Spatial scale	Landscape connectivity metric	Description
Intra-patch connectivity	Focal patch area (ha) (log-transformed)	Structural connectivity metric quantifying the area (ha) of mature forest available to a hummingbird without crossing a non-forested gap
	Focal patch area (ha) within 1 km radius (log-transformed)	Simple functional connectivity metric quantifying the area (ha) of mature forest available to a hummingbird, without crossing a non-forested gap and within their maximum daily movement range
	Distance-weighted focal patch area	Functional connectivity metric in which the contribution of mature forest area (ha) available without crossing a non-forested gap decreases with the distance from the sampling site, relative to the mean home range length of traplining hummingbirds
Local landscape connectivity	Unlimited gap-crossing: a) ‘narrow’ vs ‘broad’ habitat definition b) without or with distance weighting	Four functional connectivity metrics quantifying the trapliner habitat area (ha) available within the maximum daily movement range of traplining hummingbirds, assuming that these pollinators do not avoid crossing non-forested gaps
	Gap-crossing threshold: a) ‘narrow’ vs ‘broad’ habitat definition b) without or with distance weighting	Four functional connectivity metrics quantifying the trapliner habitat area (ha) available within the maximum daily movement range of traplining hummingbirds, without crossing any non-forested gap wider than 50 m
	Probabilistic gap-crossing: a) ‘narrow’ vs ‘broad’ habitat definition b) without or with distance weighting	Four functional connectivity metrics quantifying the trapliner habitat area (ha) available within the maximum daily movement range of traplining hummingbirds, where the contribution to landscape connectivity decreases with the summed distance of non-forested gaps that needs to be crossed

#### The incidence function model

The incidence function model (IFM) is a distance- and area-weighted approach that can be parameterized to predict the dynamics of a network of patches, including patterns of extinction and colonization for a particular species (Hanski 1994). The implementation of the IFM as a functional landscape connectivity metric ${S}_{i}$ for a focal patch *i* incorporates (Eq. 1): (1) a distance-weighted dispersal kernel that includes the distance ${d}_{ij}$ between a focal patch $i$ and each surrounding patch $j$, as well as a scaling parameter $\alpha$ which scales this distance relative to species-specific movement capacity; 2) an area-weighted measure ${A}_{j}$ of each surrounding patch $j$; and (3) other characteristics ${p}_{j}$ of each surrounding patch $j$.

1 $$S_{i} = \mathop \sum \limits_{j \ne i} e^{{ - \alpha d_{ij} }} A_{j} p_{j}$$

In its original form, based on discrete habitat areas, the functional landscape connectivity metric ${S}_{i}$ represents the degree to which a focal patch $i$ is integrated into a network of patches, and the overall distribution of the ${S}_{i}$ values across the patches in a local landscape can describe the overall functional landscape connectivity of the network of patches (Hanski 1994). Given that we used rasterized local landscapes that represent trapliner habitat, rather than a network of patches, we redefined $i$ and $j$ in Eq. 1 to represent individual pixels (*i.e.,* raster cells of 10 m by 10 m) in the local landscape and not distinct patches (Leimberger 2022). Thus, for each rasterized local landscape, $i$ represents the pixel where the sampling site is located and $j$ represents any other pixel that is considered trapliner habitat. Thus, the value of the raster-cell-based functional landscape connectivity metric ${S}_{i}$ represents the amount of trapliner habitat that a particular raster cell $i$ (*e.g.,* the location of the sampling site) is connected to.

For each rasterized local landscape, which included the ‘narrow’ and ‘broad’ definitions of trapliner habitat, we calculated the Euclidean centre-to-centre distance ${d}_{ij}$ between the sampling site pixel $i$ and each trapliner habitat pixel $j$. We used a scaling parameter $\alpha$ that represented the inverse of the mean home range length of *Phaethornis guy* (282 m), the most common traplining hummingbird in the study area and the most frequent visitor of *H. tortuosa* (Volpe et al. 2016; Leimberger et al. 2023): $\alpha =\frac{1}{282}$. While this choice allowed us to base our analysis on empirical movement behaviour data, our objective was not to optimize $\alpha$, but to provide an initial assessment of its influence on the predictive capacity of landscape connectivity metrics. Although exploring a range of $\alpha$ values could provide additional insights, this lies beyond the scope of this study and represents an avenue for future work. The trapliner habitat values (1 for habitat and 0 for non-habitat) of pixels $j$ were used as ${p}_{j}$ in Eq. 1, so that non-habitat pixels would not contribute to the ${S}_{i}$ value. Given that all pixels $j$ had an equivalent area (10 m by 10 m), we defined the parameter ${A}_{j}$ as 1. Thus, for each local landscape centered around a sampling site *i*, we used Eq. 1 to calculate the sum of the ${S}_{ij}$ values $\left(\sum_{j\ne i}{e}^{-\alpha {d}_{ij}}\right)$ for all the trapliner habitat pixels $j$ to obtain a potential functional landscape connectivity metric ${S}_{i}$, separately for the ‘narrow’ and ‘broad’ definitions of trapliner habitat.

One benefit of using rasterized local landscapes is that the ${S}_{i}$ can be estimated for the focal patch to obtain a distance-weighted landscape connectivity metric that represents the habitat available within the focal patch (*i.e.,* intra-patch connectivity). That is, when restricting the analysis to habitat pixels $j$ that belong to the focal patch, we can quantify the relative landscape connectivity of trapliner habitat available in the focal patch, as a function of the distance ${d}_{ij}$ between each habitat pixel *j* and the sampling location *i* (Eq. 1). Further, by modifying the assigned trapliner habitat value ${p}_{j}$ to represent the probability that a trapliner habitat pixel $j$ is connected to the sampling location $i$ (*e.g.,* in relation to the size of non-habitat gaps separating the two pixels), Eq. 1 can incorporate the effect of gap avoidance behaviour. This approach can be used to estimate functional connectivity at the local landscape scale, reflecting the combined contribution of all trapliner habitat areas within that landscape and the effect of non-forested gaps as a function of gap size. We estimated all the functional landscape connectivity metrics $\left({S}_{i}\right)$ in R 4.3.3. (R Core Team 2023) with the terra 1.7-74 (Hijmans 2024) and sf 1.0-16 (Pebesma & Bivand 2023) packages.

#### Intra-patch connectivity

Intra-patch connectivity represents the properties of the focal habitat patch in which a given sampling site $i$ is located. We only considered the ‘narrow’ trapliner habitat definition to delineate the focal patch, as it would not be practical to delineate discrete forest patches under the ‘broad’ habitat definition due to the connecting nature of narrow forest elements. We quantified three intra-patch connectivity metrics that differed in the way that pollinator movement behaviour was considered (Table 1).

First, following Hadley et al., (2014) and Torres‐Vanegas et al., (2021), we ignored movement distance and quantified *focal patch area* as the log-transformed area (ha) of the entire focal patch, regardless of whether it extended beyond the local landscape boundary (1 km radius). This represented a purely structural landscape connectivity metric that measures the total area of mature forest available to a hummingbird without requiring it to cross a non-forested gap. Second, we modified the first metric by imposing a distance threshold of 1 km to quantify *focal patch area within 1 km radius* as the log-transformed area (ha) of the part of each focal patch that was within a 1 km radius of the corresponding sampling site. This simple functional landscape connectivity metric represents the area (ha) of mature forest available to a hummingbird, without crossing a non-forested gap and within their maximum daily movement range.

Third, we defined *distance-weighted focal patch area* as a distance-weighted potential functional connectivity metric based on Eq. 1. This represented the probability that pollinators move from any focal patch pixel $j$ to the sampling site pixel $i$. That is, for each local landscape, we estimated the distance-weighted, negative exponential dispersal kernel for all the trapliner habitat pixels $j$ within the focal patch as: $\sum_{j\ne i}{e}^{-\alpha {d}_{ij}}$. This represents a measure of intra-patch connectivity where the contribution of a focal patch pixel $j$ is assumed to decrease with the distance ${d}_{ij}$ to the sampling site pixel $i$. Thus, an elongated patch will have a lower intra-patch connectivity value compared to a round patch of the same area, as more distant pixels contribute less to connectivity. The parameter $\alpha =\frac{1}{282}$ rescales the distance ${d}_{ij}$ relative to the mean home range length of *Phaethornis guy*, the principal pollinator of *H. tortuosa*. This *distance-weighted focal patch area* was log-transformed.

#### Local landscape connectivity

**Fig. 2:**
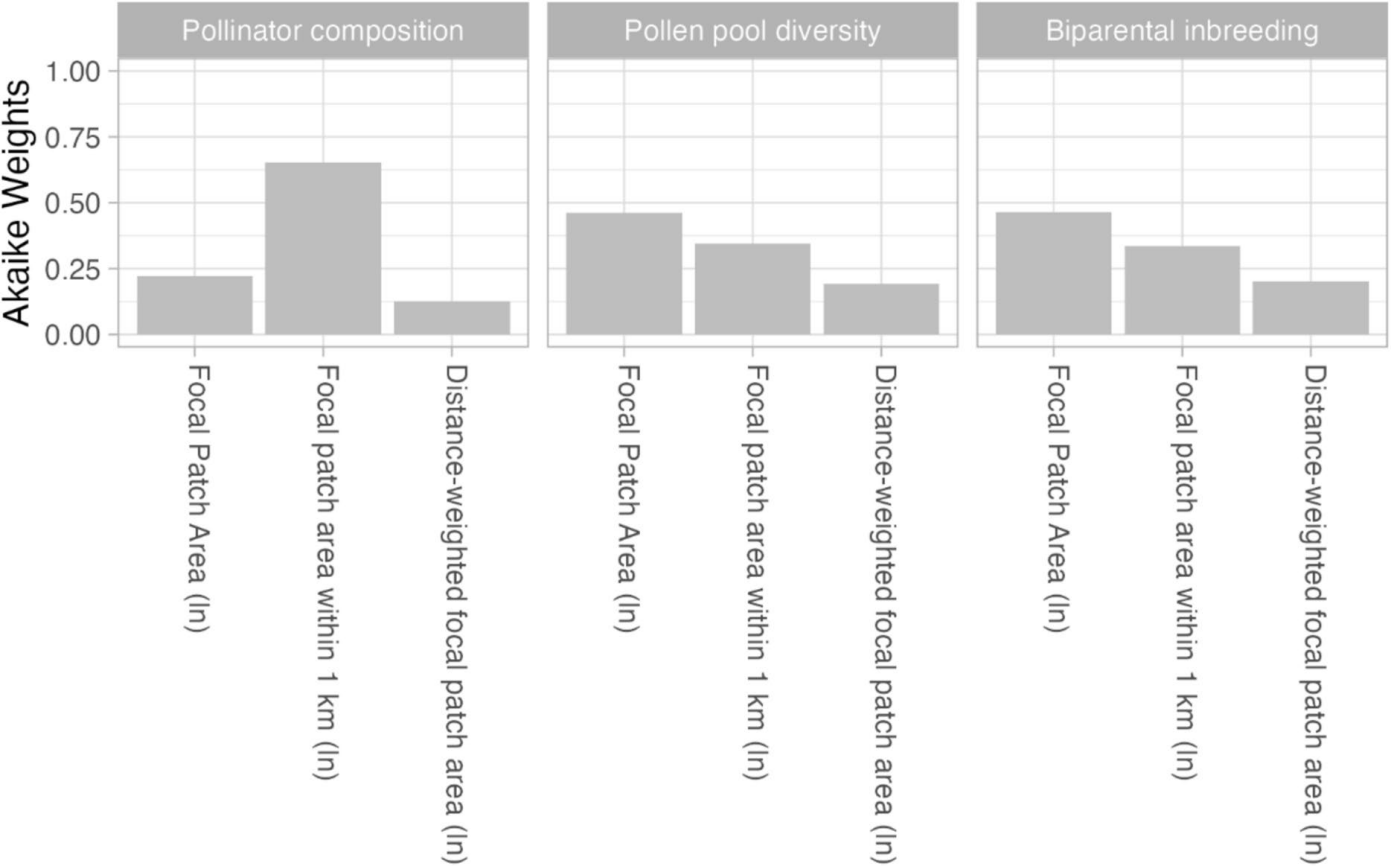
Relative importance of intra-patch connectivity metrics. Each bar shows the Akaike weight (Table 2) of a simple regression model with an intra-patch connectivity metric as a single predictor variable, separately for each response variable (represented in each facet). Within each facet, the Akaike weights sum to one. The pollinator composition corresponds to the proportion of high-mobility hummingbirds, based on the subset of 13 focal patches with hummingbird capture data. The values for the other response variables are based on the complete set of 30 focal patches

To quantify how all trapliner habitat pixels $j$ within the local landscape (including the focal patch) contribute to the overall landscape connectivity of the sampling site pixel $i$, we considered all possible combinations of three factors: (1) trapliner habitat definition (‘narrow’ or ‘broad’) (Figs. 1A-C); (2) distance-weighting as described above (unweighted or weighted) (Figs. 1G-H); and (3) gap-crossing behaviour (unlimited, threshold, or probabilistic) (Figs. 1D-F). This resulted in a total of $2\times 2\times 3=12$ local landscape connectivity metrics (Fig. 1; Table 1), where the metric with the ‘broad’ trapliner habitat definition, distance-weighting and probabilistic gap-crossing represented the most complex functional landscape connectivity metric, as it incorporates multiple dimensions of movement behaviour. We expressed each metric in units of trapliner habitat area (ha) that is functionally connected to a sampling site pixel $i$ under the assumptions of the given metric.

Metrics with unlimited gap-crossing behaviour and without distance-weighting quantified the *amount of trapliner habitat area within a 1 km radius* of each sampling site, which included the pixels of the focal patch. When using the ‘narrow’ habitat definition without distance-weighting, this is proportional to the metric *proportion of forest within 1 km* used by Hadley et al., (2014) and Torres‐Vanegas et al., (2021). It considers any trapliner habitat available, irrespective of non-forested gaps, as long as it lies within the maximum daily movement range of traplining hummingbirds.

Metrics with a *gap-crossing threshold* considered that trapliner habitat patches in the local landscape were connected if their edge-to-edge distance was ≤ 50 m. Radio-tracking of *Phaethornis guy* demonstrated that a non-forested gap of 50 m reduces the odds of crossing by approx. 50% (Volpe et al. 2014). We implemented this threshold using a 25 m buffer area around all patches in each local landscape (Fig. 1E). We considered that non-focal patches were connected to the focal patch (*i.e.,* where the sampling site pixel $i$ is located) if there was a direct overlap in the buffer areas, or if the overlap occurred indirectly through other connected non-focal patches (*i.e.,* stepping stones). Accordingly, pixels in connected habitat patches (including the focal patch) were assigned ${p}_{j}=1,$ whereas pixels in unconnected habitat patches were assigned ${p}_{j}=0$ in Eq. 1.

To define functional connectivity metrics with *probabilistic gap-crossing*, we modified ${p}_{j}$ in Eq. 1 to reflect the probability that habitat patch $k$ (which contained pixel *j*) was connected to the focal patch (which contained the sampling site pixel $i$), given the non-forested gap distance ${d}_{k}$. In case of indirect connections, we calculated ${d}_{k}$ as the minimum sum of gap distances between patch *k* and the focal patch. Volpe et al., (2014) estimated that a reduction of 50% in the odds of crossing a non-forested gap occurred between a gap distance of 46 and 63 m, based on which we chose an intermediate distance of ${d}_{k}=50 \mathrm{m}$ to correspond to a 50% reduction. Hence, we defined ${p}_{j}$ according to a negative exponential function with parameter $\lambda =\frac{\mathrm{log}\left(2\right)}{50}=0.013$ (Eq. 2).

2 $$p_{j} = e^{{ - \lambda d_{k} }}$$

The 50% gap-crossing threshold was not intended to represent an optimized parameter of pollinator movement behaviour, but rather a commonly used and interpretable reference value for defining local landscape connectivity. This value serves to contrast a binary conceptualization of local landscape connectivity, in which habitat patches are considered connected if their edge-to-edge distance is ≤ 50 m, with a *probabilistic gap-crossing* framework that allows continuous variation in gap-crossing probabilities across non-forested gaps.

### Landscape connectivity metrics and pollen-mediated gene flow

To evaluate the impact of the structural and functional components of landscape connectivity on contemporary pollen-mediated gene flow (*i.e.,* actual functional connectivity) and the composition of the pollinator community, we fitted a simple regression model for each response variable (proportion of high-mobility hummingbirds, haplotype diversity (*h*) of pollen pools, and biparental inbreeding (*t*_*m*_ – *t*_*s*_)) using the lm function in R 4.3.3 (R Core Team 2023). We applied an arcsine square-root transformation to the proportion of high-mobility hummingbirds and the haplotype diversity (*h*) of pollen pools, as these variables are constrained from zero to one. All variables were standardized (after any applicable transformation), so that the regression slope coefficients represent beta coefficients, which can be directly compared between all models.

To evaluate the relative importance of the different functional components of our landscape connectivity metrics (*i.e.,* trapliner habitat definition, distance-weighting, and gap-crossing behaviour) within a given set of models (either intra-patch connectivity or local landscape connectivity), we estimated $\Delta {AIC}_{c}$ values (small sample-size corrected $AIC$) and calculated Akaike weights based on ${AIC}_{c}$ values using the sw function of the MuMIn package (Barton 2017). Here, ${w}_{m}={e}^{-{\Delta AICc}_{m}/2}/{\sum }_{n}{e}^{-{\Delta AICc}_{n}/2}$ is the weight of model $m$ in a set of models $n.$ In addition to the sampling site-level analysis reported here, we also fitted linear mixed-effects models at the maternal plant level, which included focal patch ID as a random effect. Since this did not change the nature or interpretation of the results, we report only the models at the sampling site level.

We did consider modeling contemporary pollen-mediated gene flow as a function of multiple predictor variables (*i.e.,* one intra-patch connectivity metric and one local landscape connectivity metric, calculated with or without the focal patch). However, high levels of correlation between the metrics confounded the results (Supplementary Methods and Results). Exploration of the behaviour of the IFM in simulated landscapes showed that we could not disentangle the effect of focal patch area from local landscape connectivity (Fig. S3). We therefore opted not to pursue models with multiple predictor variables. Hence, we considered intra-patch connectivity metrics and local landscape connectivity metrics to be alternative explanations of actual functional connectivity as represented by our response variables. That is, metrics of intra-patch connectivity assume that pollinators do not cross non-forested gaps at all, and local landscape connectivity metrics assume that the same pollinator movement parameters apply to all trapliner habitat, irrespective of patch identity (*i.e.,* focal vs non-focal patch).

## Results

### Intra-patch connectivity

All intra-patch connectivity metrics (Table 1) had positive effects on the proportion of high-mobility hummingbirds (*i.e.,* composition of the pollinator community) and the haplotype diversity (*h*) of pollen pools (*i.e.,* genetic diversity), as evidenced by the consistently positive slope coefficients and standard errors that did not include zero (Table 2). In contrast, intra-patch connectivity metrics had consistently negative effects on biparental inbreeding (*t*_*m*_—*t*_*s*_), as evidenced by the negative slope coefficients (Table 2). These findings align with the expectation that larger habitat areas (*i.e.,* intra-patch connectivity) promote pollen transfer by supporting greater availability of floral resources and more frequent pollinator visits, thereby enhancing genetic diversity and reducing the incidence of inbreeding.

**Table 2:** Model comparison for intra-patch connectivity metrics, separately for each response variable. For each metric, summary results of a simple linear regression model are shown. The slope coefficients are beta coefficients, shown with their standard error (SE). The *R*^*2*^ refers to the unadjusted coefficient of determination and the *AIC*_*C*_ indicates the Akaike information criterion with sample size correction. The *ΔAIC*_*C*_ represents the difference between a model’s *AIC*_*C*_ value and the lowest *AIC*_*C*_ value for the same response variable. The model weights *w*_*m*_ represents the relative support for each of the three intra-patch connectivity metrics for the same response variable. The values for the proportion of high-mobility hummingbirds are based on the subset of 13 focal patches with hummingbird capture data, whereas the values for the other response variables are based on the complete set of 30 focal patches. The best-supported model (*i.e.,* lowest *AIC*_*C*_ value) for each response variable is presented in bold

Response variable	Distance limitation	Landscape connectivity metric	Slope ± SE	*R*^*2*^	*AIC*_*C*_	*ΔAIC*_*C*_	*w*_*m*_
Proportion of high-mobility hummingbirds	Unlimited	Focal patch area	0.617 ± 0.231	0.392	38.05	2.17	0.221
	**Threshold 1 km**	**Focal patch area within 1 km radius**	**0.781 ± 0.242**	**0.486**	**35.88**	**0.00**	**0.654**
	Weighted	Distance-weighted focal patch area	0.698 ± 0.295	0.340	39.19	3.31	0.125
Haplotype diversity (*h*) of pollen pools	**Unlimited**	**Focal patch area**	**0.470 ± 0.167**	**0.221**	**83.57**	**0.00**	**0.462**
	Threshold 1 km	Focal patch area within 1 km radius	0.453 ± 0.168	0.205	84.15	0.58	0.345
	Weighted	Distance-weighted focal patch area	0.417 ± 0.172	0.174	85.32	1.75	0.192
Biparental inbreeding (*t*_*m*_—*t*_*s*_)	**Unlimited**	**Focal patch area**	**− 0.373 ± 0.175**	**0.139**	**86.56**	**0.00**	**0.466**
	Threshold 1 km	Focal patch area within 1 km radius	− 0.346 ± 0.177	0.120	87.22	0.66	0.334
	Weighted	Distance-weighted focal patch area	− 0.298 ± 0.180	0.089	88.25	1.69	0.200

When comparing models with the same response variable, we considered models with $\Delta {AIC}_{c}>2$ as less supported by the data. The composition of the pollinator community was best predicted by *focal patch area within 1 km radius*, as indicated by the corresponding lowest ${AIC}_{C}$ value (Table 2) and highest Akaike weights (Fig. 2). In contrast, the two alternative intra-patch connectivity metrics, *focal patch area* ($\Delta {AIC}_{C}=2.17$) and *distance-weighted focal patch area* ($\Delta {AIC}_{C}=3.31$) were less supported (Fig. 2; Table 2).

For genetic diversity and biparental inbreeding, the three intra-patch connectivity metrics (Table 1) showed similar statistical support $\left(\Delta {AIC}_{C}<2\right)$, indicating that no single model could be identified as best-supported (Table 2). The Akaike weights were somewhat higher for *focal patch area* and lowest for *distance-weighted focal patch area* (Table 2). When restricting the analysis of the genetic metrics to the sampling sites with hummingbird capture data, *distance-weighted focal patch area* was less supported (Fig. S4; Table S1).

### Local landscape connectivity

**Fig. 3:**
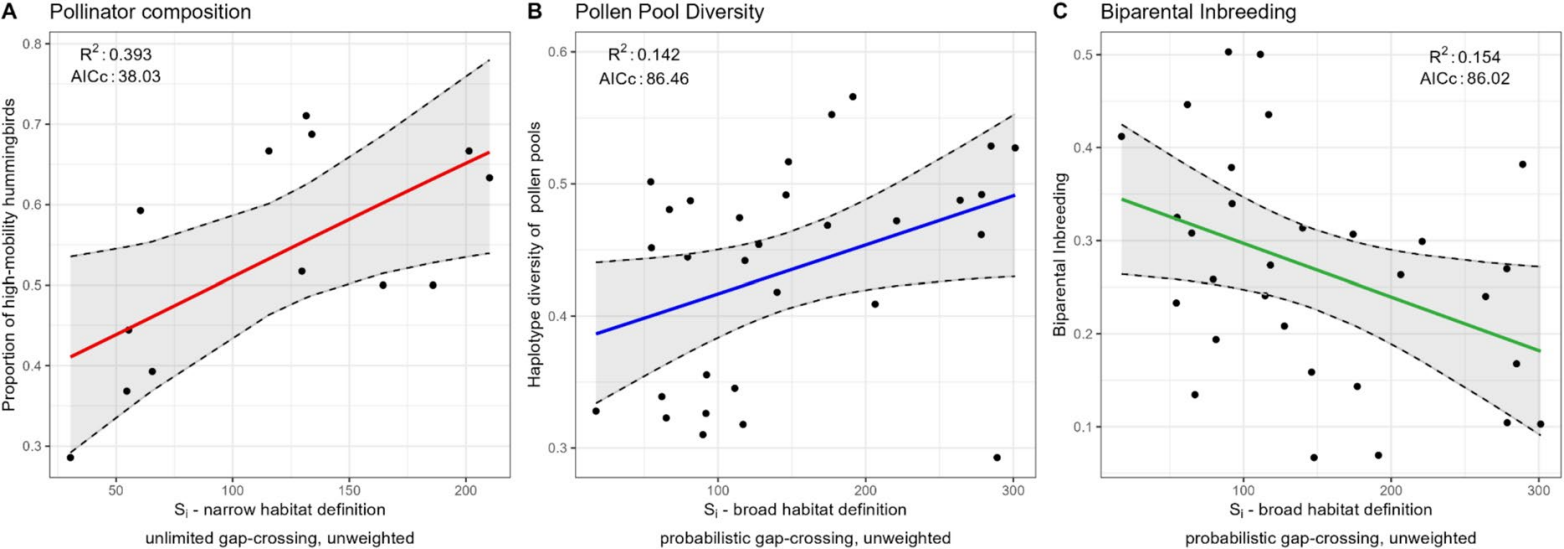
Relationships between local landscape connectivity metrics and ecological response variables: **A** the proportion of high-mobility hummingbirds; **B** the haplotype diversity (*h*) of pollen pools; and **C** biparental inbreeding (*t*_*m*_*—t*_*s*_). Each panel includes the local landscape connectivity metric that best explained variation in each response variable, as determined by the lowest ${AIC}_{C}$ value (Table 3). The solid lines represent predicted values generated from the fitted models across the observed range of each predictor variable, and the dashed lines represent the 95% confidence intervals of the predicted values. The points correspond to the observed empirical data: values for the proportion of high-mobility hummingbirds are based on the subset of 13 focal patches with hummingbird capture data, whereas the values for the other response variables are based on the complete set of 30 focal patches

All local landscape connectivity metrics (Table 1) had positive effects on the proportion of high-mobility hummingbirds (*i.e.,* composition of the pollinator community) and the haplotype diversity (*h*) of pollen pools (*i.e.,* genetic diversity), and negative effects on biparental inbreeding (*t*_*m*_—*t*_*s*_) (Table 3; Figs. 3, S5-S7). These findings support the expectation that greater local landscape connectivity promotes pollen transfer by facilitating pollinator movement across the landscape, increasing the frequency of inter-patch visits, thereby enhancing genetic diversity and reducing the incidence of inbreeding.

**Table 3:** Model comparison for local landscape connectivity metrics, separately for each response variable. For each metric (row), summary results of a simple linear regression model are shown. The slope coefficients are beta coefficients, shown with their standard error (SE). The *R*^*2*^ refers to the unadjusted coefficient of determination and the *AIC*_*C*_ indicates the Akaike information criterion with sample size correction. The *ΔAIC*_*C*_ represents the difference between a model’s *AIC*_*C*_ value and the lowest *AIC*_*C*_ value for the same response variable. The model weights *w*_*m*_ represents the relative support for each of the twelve local landscape connectivity metrics for the same response variable. The values for the proportion of high-mobility hummingbirds are based on the subset of 13 focal patches with hummingbird capture data, whereas the values for the other response variables are based on the complete set of 30 focal patches. The best-supported model (*i.e.,* lowest *AIC*_*C*_ value) for each response variable is presented in bold

Response variable	Habitat definition	Distance weighting	Gap-crossing	Slope ± SE	*R*^*2*^	*AICc*	*ΔAIC*_*C*_	*w*_*m*_
Proportion of high-mobility hummingbirds	**Narrow**	**Unweighted**	**Unlimited**	**0.513 ± 0.192**	**0.393**	**38.03**	**0.00**	**0.187**
			Threshold	0.496 ± 0.200	0.359	38.73	0.71	0.131
			Probabilistic	0.459 ± 0.187	0.354	38.84	0.81	0.125
		Weighted	Unlimited	0.431 ± 0.185	0.330	39.32	1.29	0.098
			Threshold	0.451 ± 0.204	0.309	39.72	1.69	0.080
			Probabilistic	0.441 ± 0.199	0.308	39.72	1.69	0.080
	Broad	Unweighted	Unlimited	0.519 ± 0.274	0.246	40.86	2.83	0.045
			Threshold	0.536 ± 0.270	0.264	40.54	2.51	0.053
			Probabilistic	0.518 ± 0.242	0.295	39.98	1.95	0.070
		Weighted	Unlimited	0.472 ± 0.249	0.246	40.85	2.82	0.046
			Threshold	0.440 ± 0.254	0.214	41.38	3.36	0.035
			Probabilistic	0.469 ± 0.241	0.255	40.68	2.66	0.049
Haplotype diversity (*h*) of pollen pools	Narrow	Unweighted	Unlimited	0.157 ± 0.157	0.025	90.29	3.83	0.037
			Threshold	0.051 ± 0.051	0.003	90.96	4.50	0.026
			Probabilistic	0.136 ± 0.136	0.018	90.48	4.02	0.034
		Weighted	Unlimited	0.100 ± 0.100	0.010	90.74	4.28	0.030
			Threshold	0.044 ± 0.044	0.002	90.98	4.52	0.026
			Probabilistic	0.090 ± 0.090	0.008	90.80	4.34	0.029
	**Broad**	**Unweighted**	Unlimited	0.242 ± 0.183	0.059	89.23	2.77	0.063
			Threshold	0.250 ± 0.183	0.063	89.10	2.64	0.067
			**Probabilistic**	**0.376 ± 0.175**	**0.142**	**86.46**	**0.00**	**0.251**
		Weighted	Unlimited	0.283 ± 0.181	0.080	88.53	2.07	0.089
			Threshold	0.320 ± 0.179	0.102	87.80	1.34	0.129
			Probabilistic	0.366 ± 0.176	0.134	86.73	0.27	0.220
Biparental inbreeding (*t*_*m*_—*t*_*s*_)	Narrow	Unweighted	Unlimited	−0.224 ± 0.184	0.050	89.50	3.48	0.043
			Threshold	−0.156 ± 0.187	0.024	90.30	4.28	0.029
			Probabilistic	−0.157 ± 0.187	0.025	90.29	4.27	0.029
		Weighted	Unlimited	−0.140 ± 0.187	0.019	90.45	4.43	0.027
			Threshold	−0.083 ± 0.188	0.007	90.83	4.81	0.022
			Probabilistic	−0.088 ± 0.188	0.008	90.81	4.79	0.022
	**Broad**	**Unweighted**	Unlimited	−0.288 ± 0.181	0.083	88.45	2.43	0.073
			Threshold	−0.291 ± 0.181	0.085	88.39	2.37	0.075
			**Probabilistic**	**−0.393 ± 0.174**	**0.154**	**86.02**	**0.00**	**0.244**
		Weighted	Unlimited	−0.319 ± 0.179	0.102	87.83	1.81	0.099
			Threshold	−0.351 ± 0.177	0.124	87.09	1.06	0.144
			Probabilistic	−0.375 ± 0.175	0.141	86.49	0.46	0.194

The composition of the pollinator community was best explained by the *amount of trapliner habitat area within a 1 km radius*, as indicated by the lowest ${AIC}_{C}$ value (Fig. 3; Table 3). This corresponds to the simplest functional landscape connectivity metric, which only considers the amount of mature forest available in the local landscape without further restrictions on pollinator movement within or between patches (Table 1). In contrast, genetic diversity and biparental inbreeding were best explained (*i.e.,* lowest ${AIC}_{C}$ value) by the two functional landscape connectivity metrics that made the most detailed assumptions about pollinator movement behaviour (Table 3). These metrics both incorporated the ‘broad’ definition of trapliner habitat and *probabilistic gap-crossing* behaviour, but differed in whether distance-weighting was included or not (Fig. 3; Table 3).

**Fig. 4:**
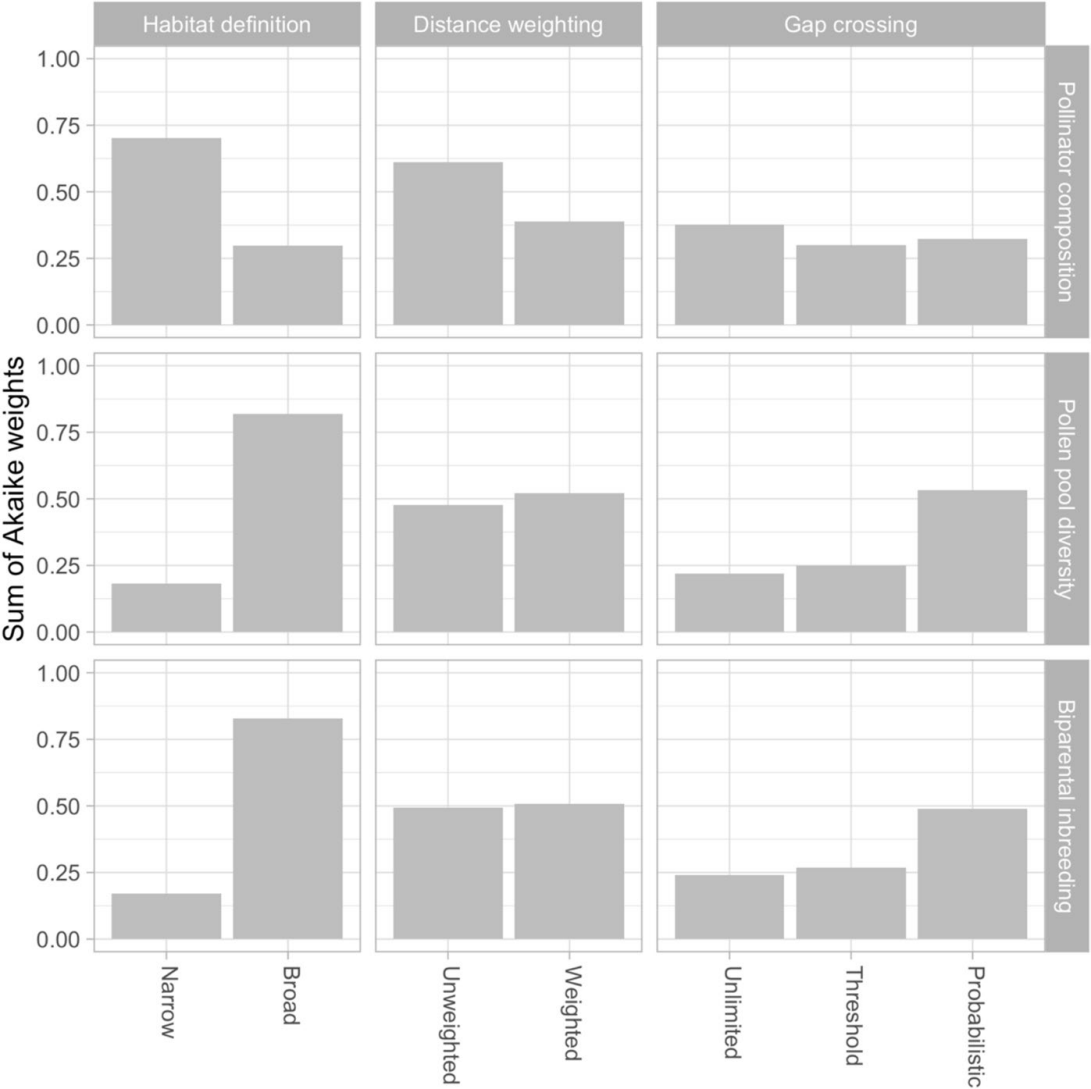
The summed Akaike weights for local landscape connectivity metrics. The three columns facets represent the three factors that modified different aspects of functional landscape connectivity (*i.e.,* trapliner habitat definition, distance-weighting, and gap-crossing behaviour). The three response variables are represented in rows. Each bar shows the sum of the Akaike weights of all local landscape connectivity metrics that included the respective factor level (*i.e.,* sum over six models for two-level factors or sum over four models for three-level factors). Within each facet, bar heights sum to one, and the height of a bar indicates the empirical support for a factor level. The pollinator composition corresponds to the proportion of high-mobility hummingbirds, based on the subset of 13 focal patches with hummingbird capture data. The values for the other response variables are based on the complete set of 30 focal patches

Local landscape connectivity metrics based on the ‘narrow’ definition of trapliner habitat (*i.e.,* mature forest) tended to better explain variation in the composition of the pollinator community compared to those based on the ‘broad’ definition of trapliner habitat (*i.e.,* mature forest, regenerating forest, and narrow forest elements), most of which showed $\Delta {AIC}_{C}>2$ values (Table 3) and lower Akaike weights (Fig. 4). In contrast, variation in genetic diversity and biparental inbreeding was best explained by local landscape connectivity metrics that included the ‘broad’ definition of trapliner habitat. This included forest elements, which are expected to facilitate pollinator movement beyond mature forest, whereas all models with the ‘narrow’ habitat definition showed $\Delta {AIC}_{C}>2$ values (Table 3) and lower Akaike weights (Fig. 4).

Variation in the composition of the pollinator community showed a tendency to be better predicted by local landscape connectivity metrics that did not include distance-weighting, although $\Delta {AIC}_{C}$ values were generally small among weighted and unweighted metrics (Fig. 3; Table 3). This response variable was equally well predicted by models that integrated distinct types of gap-crossing behaviour, as indicated by similar ${\Delta AIC}_{C}$ values (Table 3) and Akaike weights (Fig. 4). In contrast, for models based on the ‘broad’ definition of trapliner habitat, genetic diversity and biparental inbreeding tended to be better predicted by local landscape connectivity metrics that included a *probabilistic gap-crossing* behaviour, compared to those that included a *gap-crossing threshold* or *unlimited gap-crossing* (Fig. 4; Table 3). These metrics represented the probability that a non-focal patch in the local landscape was connected to the focal patch, based on the likelihood that a traplining hummingbird is expected to cross a non-forested gap. This represents the most functionally realistic landscape connectivity metric, as it incorporates a gap-crossing probability that declines with increasing distance across non-forested gaps. Genetic diversity and biparental inbreeding indicated no clear preference regarding distance-weighting (Fig. 4; Table 3).

The nature of these differences between the composition of the pollinator community and genetic diversity and biparental inbreeding did not change substantially when restricting the analysis to the subset of focal patches with hummingbird capture data (Fig. S8; Table S2). However, the ${\Delta AIC}_{C}$ values were generally lower (which may reflect lower statistical power due to the smaller sample size), meaning that the differences in model performance ($\Delta {AIC}_{C}$) between models using different landscape connectivity metrics as predictors were less pronounced.

## Discussion

In this study, we adapted the incidence function model (IFM) to develop fine-scale landscape connectivity metrics that represented potential functional landscape connectivity for a hummingbird-pollinated plant. These metrics integrated empirically collected data on movement behaviour of traplining hummingbirds (*e.g.,* mean home range length and gap-crossing probability) with detailed spatial data describing landscape structure. We assessed contemporary pollen-mediated gene flow as a measure of actual functional landscape connectivity through pollination. Our approach enabled us to quantify both intra-patch and local landscape connectivity (Table 1), and to compare their ability to predict the composition of the local pollinator community and contemporary pollen-mediated gene flow (Table 2; Table 3). Within a focal patch, pollen transport is primarily driven by structural landscape connectivity and is less explained by the distance-weighted intra-patch connectivity metric. Local landscape connectivity metrics that included the ‘broad’ definition of trapliner habitat and a gap-crossing probability function better predicted contemporary pollen-mediated gene flow than connectivity metrics including a ‘narrow’ habitat definition or uninformed by pollinator movement behaviour. This highlights the importance of suboptimal habitat areas for pollinator movement and pollen-mediated gene flow, indicating that a more nuanced representation of hummingbird movement more accurately represents functional landscape connectivity in *H. tortuosa*. More broadly, our results emphasize the importance of regenerating forests and narrow forest elements for hummingbird movement and pollen transfer in fragmented tropical landscapes.

### Intra-patch connectivity

When focusing on intra-patch connectivity metrics (Table 1), we found that the genetic metrics were better explained by *focal patch area* (Fig. 2; Table 2), though the two more functional metrics also received empirical support. In contrast, the proportion of high-mobility hummingbirds was best predicted by *focal patch area within 1 km radius* (Fig. 2; Table 2). The strong support for this intra-patch connectivity metric suggests that limiting the area considered to be within the maximum daily movement range of traplining hummingbirds (Volpe et al. 2016) is sufficient to capture biologically relevant distance limitations, as it likely reflects key aspects of the foraging behaviour and habitat use of hummingbirds. Contiguous mature forest is a logical determinant of hummingbird availability, as it enables them to forage efficiently and enhances pollen transport without the need to cross a non-forested gap (Hadley et al. 2014; Torres‐Vanegas et al. 2021; Jones et al. 2022). In our study system, traplining hummingbirds prefer to forage within large and contiguous patches with dense floral resources (Volpe et al. 2014; Hadley et al. 2018; Torres‐Vanegas et al. 2021; Jones et al. 2022). These habitat areas facilitate the ability of traplining hummingbirds to forage across long-distance routes, provide nectar that satisfies their energetic demands, and reduce interspecific competition with territorial hummingbirds (Hadley & Betts 2009, 2012; Auffret et al. 2017).

The distance-weighted intra-patch connectivity metric assumes that the probability of hummingbird movement decreases with the distance between the sampling site and any other point within the focal patch. This implies that even at a reduced spatial scale, hummingbird movement within a focal patch would incur an energetic cost, regardless of the availability of floral resources. However, this may not reflect biological reality in our study system, as traplining hummingbirds typically forage across long-distance routes (up to 1 km routes multiple times per day) to acquire nectar (Volpe et al. 2014; Betts et al. 2015) and may not perceive movement at fine spatial scales as costly (Volpe et al. 2016; Leimberger et al. 2023). The lack of support for the distance-weighted intra-patch connectivity metric suggests that contiguous mature forest presents little to no movement cost to hummingbirds or to pollen-mediated gene flow. In our study system, pollen transport within a focal patch is primarily driven by structural landscape connectivity, as this component is directly related to hummingbird availability and nectar resources, which do not necessarily respond to our distance-weighted intra-patch connectivity metric.

In contrast, plant species that depend on pollinators with a limited movement capacity or with territorial behaviour may be more likely to experience a dramatic decline in pollen transport at a reduced spatial scale, making distance-weighted intra-patch connectivity metrics more relevant (Castilla et al. 2017; O’Connell et al. 2018). Indeed, several studies focusing on contemporary pollen-mediated gene flow have found that vertebrate-pollinated plants tend to have less genetic differentiation with increasing distance compared to invertebrate-pollinated plants (Kramer et al. 2011; Wessinger 2020; Dellinger et al. 2022).

### Definitions of trapliner habitat

When focusing on local landscape connectivity metrics (Table 1), we found that their capacity to predict the composition of the pollinator community and contemporary pollen-mediated gene flow depended on how trapliner habitat was defined (*i.e.,* ‘narrow’ vs. ‘broad’). Specifically, the composition of the pollinator community was best explained by the *amount of trapliner habitat area within a 1 km radius*, based on the ‘narrow’ definition of trapliner habitat (*i.e.,* mature forest) (Fig. 4; Table 3). In contrast, the genetic metrics were best predicted by local landscape connectivity metrics based on the ‘broad’ definition of trapliner habitat (*i.e.,* mature forest, regenerating forest, and narrow forest elements) (Fig. 4; Table 3).

While the most common traplining hummingbirds in our study system are habitat specialists and prefer mature forest with greater floral resources and better nesting sites, they also exploit other landcover types, including regenerating forest and narrow forest elements (Hadley & Betts 2009; Kormann et al. 2016). Although these landcover types may not contain high floral resource density, they can support hummingbird movement and help maintain pollen transport across the landscape (Volpe et al. 2014; Klaus et al. 2015; Gannon et al. 2021). The greater importance of mature forest (‘narrow’ definition of trapliner habitat) in explaining the composition of the pollinator community may be explained by processes other than foraging that cause traplining hummingbirds to spend more time in mature forest patches (Volpe et al. 2014; Hadley et al. 2018; Torres‐Vanegas et al. 2021; Jones et al. 2022). Mature forests provide a diversity of structural elements, including dense canopies and complex understory vegetation, which offer critical nesting sites and shelter opportunities for habitat-specialist hummingbirds (Leimberger et al. 2022). Although in some study systems the preferred habitat may not correspond to the least hindering habitat for movement (Stevens et al. 2005, 2006), in our study system mature forest is both the preferred habitat and among the least obstructive habitats for traplining hummingbirds (Hadley & Betts 2009; Volpe et al. 2014, 2016). However, decoupling habitat preference from movement resistance is an important conceptual consideration for future work in other study systems.

However, the fact that local landscape connectivity metrics with the ‘broad’ definition of trapliner habitat better predicted contemporary pollen-mediated gene flow highlights the significance of suboptimal, disturbed, or recovering habitat areas, as these landcover types contribute to a mosaic of conditions that hummingbirds exploit to fulfill their life requirements. For example, habitat-specialist hummingbirds may nest in mature forest, yet other landcover types can be important to support their long-distance foraging routes (Hadley & Betts 2009; Klaus et al. 2015; Kormann et al. 2016). Thus, the local landscape connectivity metrics that incorporate a ‘broad’ definition of trapliner habitat may align more closely with how habitat-specialist hummingbirds perceive and move across the landscape, and for this reason better predict contemporary pollen-mediated gene flow in *H. tortuosa*. Given the detailed ecological data available for this study system, future work could develop habitat suitability or resistance surfaces to incorporate continuous variation in movement resistance across mature forest, regenerating forest, narrow forest elements, arable land, and pasture.

Our results highlight the role of regenerating forest in sustaining contemporary pollen-mediated gene flow and underscore the need to distinguish recent dispersal events from patterns of standing genetic variation in adult plants. For example, Jones et al., (2022) found higher inbreeding for adult *H. tortuosa* in regenerating forests and that this effect was further exacerbated when the surrounding landscape lacked mature forest. In contrast, we found that our local landscape connectivity metrics incorporating a ‘broad’ definition of trapliner habitat in the surrounding landscape better predicted genetic diversity and biparental inbreeding (Table 3). This suggests that reduced genetic diversity in adult plants may be a product of the historical landscape, prior to the regeneration of forests that we see today. Disentangling the influence of historical and contemporary landscapes on gene flow is a challenge for the field of landscape genetics, but particularly for perennial plants, where the genetics of established individuals represent the movement of pollen and seeds in a past landscape (Sork et al. 1999; Epps & Keyghobadi 2015).

### Gap-crossing behaviour

The assumptions about pollinator gap-crossing behaviour (Table 1) had a substantial influence on the capacity of local landscape connectivity metrics to predict contemporary pollen-mediated gene flow. Among the local landscape connectivity metrics that we defined, those that included a ‘broad’ definition of trapliner habitat and a probabilistic gap-crossing function were best-supported (Fig. 4; Table 3). This indicates that the main function of suboptimal trapliner habitat (*e.g.,* regenerating forest and narrow forest elements) may lie in reducing the size of non-forested gaps and increasing the probability of pollinator movement and pollen transfer among forest patches (Mayhew et al. 2019; Rosenfield et al. 2023). The support for the probabilistic gap-crossing function is consistent with previous studies (*e.g.,* translocation experiments) showing that traplining hummingbirds are able to follow least-cost routes that avoid the non-forested matrix (Hadley & Betts 2009; Volpe et al. 2014, 2016), as this local landscape connectivity metric reduces the odds of crossing a non-forested gap as gap distance increases, constraining hummingbirds to prefer contiguous habitat that minimizes energetic costs. This suggests that it is not the amount but the spatial configuration of such habitat that matters most for the maintenance of pollen-mediated gene flow.

The improved performance of local landscape connectivity metrics with gap-crossing probability functions in predicting contemporary pollen-mediated gene flow (Fig. 4; Table 3) suggests that they more accurately represent functional landscape connectivity, as they better reflect how habitat-specialist hummingbirds interact with their environment and influence pollen transfer across the landscape. Compared to a gap-crossing threshold, a gap-crossing probability function provides a more nuanced and realistic representation of traplining hummingbird movement across the landscape, as it accounts for the gradual decline in the likelihood of crossing a non-forested gap, rather than assuming that patches are either connected or disconnected based on a fixed distance threshold (Awade & Metzger 2008; Volpe et al. 2014). It also accounts for some uncertainty, allowing that some degree of movement may be possible at larger distances, even if the probability is lower. Although it is useful to conceptualize distance thresholds to describe hummingbird movement across habitat areas, landscape connectivity does not end abruptly and is rarely all-or-nothing. Thus, a gap-crossing probability function can account for how hummingbird movement responds to the distance of non-forested gaps, and better represent the complex decision-making process that determines how hummingbirds move across the landscape (Hadley & Betts 2009; Volpe et al. 2014).

### Distance-weighting dependency

We found that weighting all raster cells within the local landscapes based on their distance to each sampling site (*i.e.,* relative to the mean home range length of *Phaethornis guy*) did not improve the capacity of local landscape connectivity metrics to predict contemporary pollen mediated gene flow (Fig. 4; Table 3). Although this distance-weighting is intended to represent the decreased likelihood of pollinator movement as distance to the sampling site increases, the application of the 1 km limit to the local landscape appears to adequately capture distance restrictions. This is based on the maximum daily movement distance of traplining hummingbirds (Betts et al. 2015; Volpe et al. 2016), which are highly mobile and move efficiently at a broad spatial scale. Consequently, the size of non-forested gaps between potential habitat patches (that include both mature and regenerating forests) appears to play a larger role in functional landscape connectivity of these pollinator species than distance alone.

In the study area, Torres-Vanegas et al., (2023) found low levels of correlated paternity within the fruits of *H. tortuosa* (approx. 0.2), so that any two seeds from the same fruit have a probability of approx. 0.8 of being sired by pollen from distinct donors. This indicates that a mixed pollen load often arrives at the flowers of *H. tortuosa*, either when a single pollinator deposits pollen from multiple donors (*i.e.,* pollen carry-over) or when successive visits of distinct pollinators deposit pollen from different donors (Torres-Vanegas et al. 2023). In either case, this is likely to result in pollen from multiple and distant donors arriving at flowers, thus weakening the decay of the pollen dispersal kernel with distance. It is possible that pollinators with different movement behaviours (*e.g.,* territorial foraging) would cause a stronger signal of distance-decay and stronger support for distance-weighting.

### Implications for the conservation of animal-mediated gene flow

In an era of prevalent habitat loss and fragmentation, conserving contemporary pollen-mediated gene flow in tropical ecosystems requires recognizing the value of a mosaic of landcover types, rather than focusing exclusively on the protection of mature forest. While mature forests often provide optimal conditions for habitat-specialist hummingbirds (*e.g.,* floral resources and nesting sites), our findings suggest that regenerating forests and narrow forest elements also support hummingbird movement and play a critical role in maintaining pollen transport (Hadley et al. 2018; Leimberger et al. 2022; Huh et al. 2023; Kormann et al. 2016). Indeed, these suboptimal or disturbed habitats have been shown to act as movement corridors or stepping stones that enable hummingbirds to forage over long distances and sustain pollen transport (Hadley & Betts 2012; Volpe et al. 2014; Kormann et al. 2016). Therefore, conservation efforts should broaden habitat definitions to include regenerating forests, living fencerows, and riparian strips, acknowledging their importance in supporting hummingbird movement and pollen transfer.

We argue that conservation efforts should focus on enhancing functional landscape connectivity, rather than just structural connectivity. Landscape connectivity models that included a gap-crossing probability function, accounting for the gradual decline in the likelihood of crossing non-forested gaps, more accurately predicted the pollen pool diversity and biparental inbreeding. A gap-crossing probability function offers insights into how contemporary pollen-mediated gene flow is shaped not only by the distance between patches, but also by the ability of hummingbirds to navigate complex and fragmented environments (Awade & Metzger 2008; Volpe et al. 2014). This underscores the importance of considering not just the amount and spatial configuration of distinct habitat areas in the landscape, but also how pollinators perceive and interact with their surroundings.

To conserve contemporary pollen-mediated gene flow, restoration and land-use planning should aim to enhance the ability of traplining hummingbirds to move across the landscape, for example, by planting shrubs, maintaining hedgerows, and preserving regenerating forests. Although these forest elements may not provide a high density of floral resources, they can facilitate hummingbird movement and support their long-distance foraging routes (Hadley & Betts 2009; Volpe et al. 2014; Kormann et al. 2016). These forest elements are particularly important in highly disturbed tropical ecosystems, where large and contiguous mature forests are lacking (Zahawi et al. 2015). Although the preservation of mature forests is essential for conservation of contemporary pollen-mediated gene flow, conservation strategies that focus solely on preserving high-quality habitat areas may fall short of maintaining pollen transfer across fragmented landscapes. Instead, an approach that includes regenerating forests and narrow forest elements, alongside a nuanced understanding of pollinator movement, will be more effective for conserving both pollinators and the plants they interact with.

## Conclusions

This study underscores the importance of incorporating pollinator-specific ecological traits into landscape connectivity models to understand contemporary pollen-mediated gene flow. We further demonstrate the value of using contemporary pollen-mediated gene flow to assess actual functional landscape connectivity in tropical plant species and provide a novel way of incorporating pollinator movement behaviour into functional landscape connectivity metrics. By parameterizing landscape connectivity metrics with field estimates of hummingbird movement behaviour, specifically home range length and gap-crossing probability, we disentangled the relative importance of the structural and functional components of landscape connectivity as determinants of contemporary pollen-mediated gene flow in *H. tortuosa*. We provide a more realistic representation of pollinator movement across fragmented landscapes by accounting for gradual declines in movement probability across non-forested gaps. Importantly, local landscape connectivity metrics incorporating a ‘broad’ definition of trapliner habitat, which included regenerating forests and narrow forest elements, outperformed those limited to mature forest. These findings highlight the role of recovering or suboptimal habitat types in maintaining pollinator movement and pollen transfer, and thus their contribution to sustaining contemporary pollen-mediated gene flow in fragmented tropical landscapes.

## Supplementary Information

Below is the link to the electronic supplementary material.

Supplementary file1 (DOCX 2744 KB)

## Data Availability

The data generated for the study and corresponding code are available in the Dryad Digital Repository 10.5061/dryad.3ffbg7b08.
