# Supplementary material for: Incorporating pollinator movement into connectivity models predicts pollen-mediated gene flow and highlights the importance of regenerating forests in tropical landscapes
Source: Landsc Ecol. 2026 Feb 14;41(3):49. doi: 10.1007/s10980-026-02309-y (PMC12971756; doi:10.1007/s10980-026-02309-y)
Supplement: Supplementary file 1 — Supplementary file1 (DOCX 2744 KB) [file 10980_2026_2309_MOESM1_ESM.docx]

**Supporting Information**

**Article title:** Incorporating hummingbird movement behaviour into landscape connectivity models predicts pollen-mediated gene flow in a fragmented tropical landscape

**Authors:** Kathryn E.C. Davis, Emil Sloth Thomassen, Helene H. Wagner, Urs G. Kormann, Adam S. Hadley, Matthew G. Betts, Felipe Torres-Vanegas

**The following Supporting Information is available for this article:**

**Supplementary Figure S1:** Each local landscape (focal forest patch 10, 12, 15, 16, 19, 22, 23, 24, 27, 28, 29, 31, 32, 33*,* and 35) that surrounded each sampling site*, defined* as the area within a 1 km radius of the median GPS coordinate of the sampled maternal plants.

**Supplementary Figure S2:** Each local landscape (focal forest patch 36, 37, 38, 39, 40, 49, 50, 52, 55, 56, 58, 59, 60, 62*,* and 63) that surrounded each sampling site*, defined* as the area within a 1 km radius of the median GPS coordinate of the sampled maternal plants.

**Supplementary Methods and Results:** Landscape Connectivity Models with Multiple Predictors – Correlations and Simulations

**Supplementary Figure S3:** Graphs depicting the relationship between focal patch area and distance-weighted $S_{i}$ metrics.

**Supplementary Figure S4:** Relative importance of intra-patch connectivity metrics for those patches with hummingbird capture data (*n* = 13).

**Supplementary Figure S5:** Relationships between local landscape connectivity metrics and the proportion of high-mobility hummingbirds.

**Supplementary Figure S6:** Relationships between local landscape connectivity metrics and the haplotype diversity (*h*) of pollen pools.

**Supplementary Figure S7:** Relationships between local landscape connectivity metrics and biparental inbreeding (*t_m_ - t_s_*).

**Supplementary Figure S8:***.* Summed Akaike weights for local landscape connectivity metrics, for those patches with hummingbird capture data (*n* = 13).

**Supplementary Table S1:** Model comparison for intra-patch connectivity metrics calculated for the *n* = 13 patches with hummingbird capture data, separately for each genetic response variable.

**Supplementary Table S2:** Model comparison for local landscape connectivity metrics calculated for the *n* = 13 patches with hummingbird *capture* data, separately for each genetic response variable.


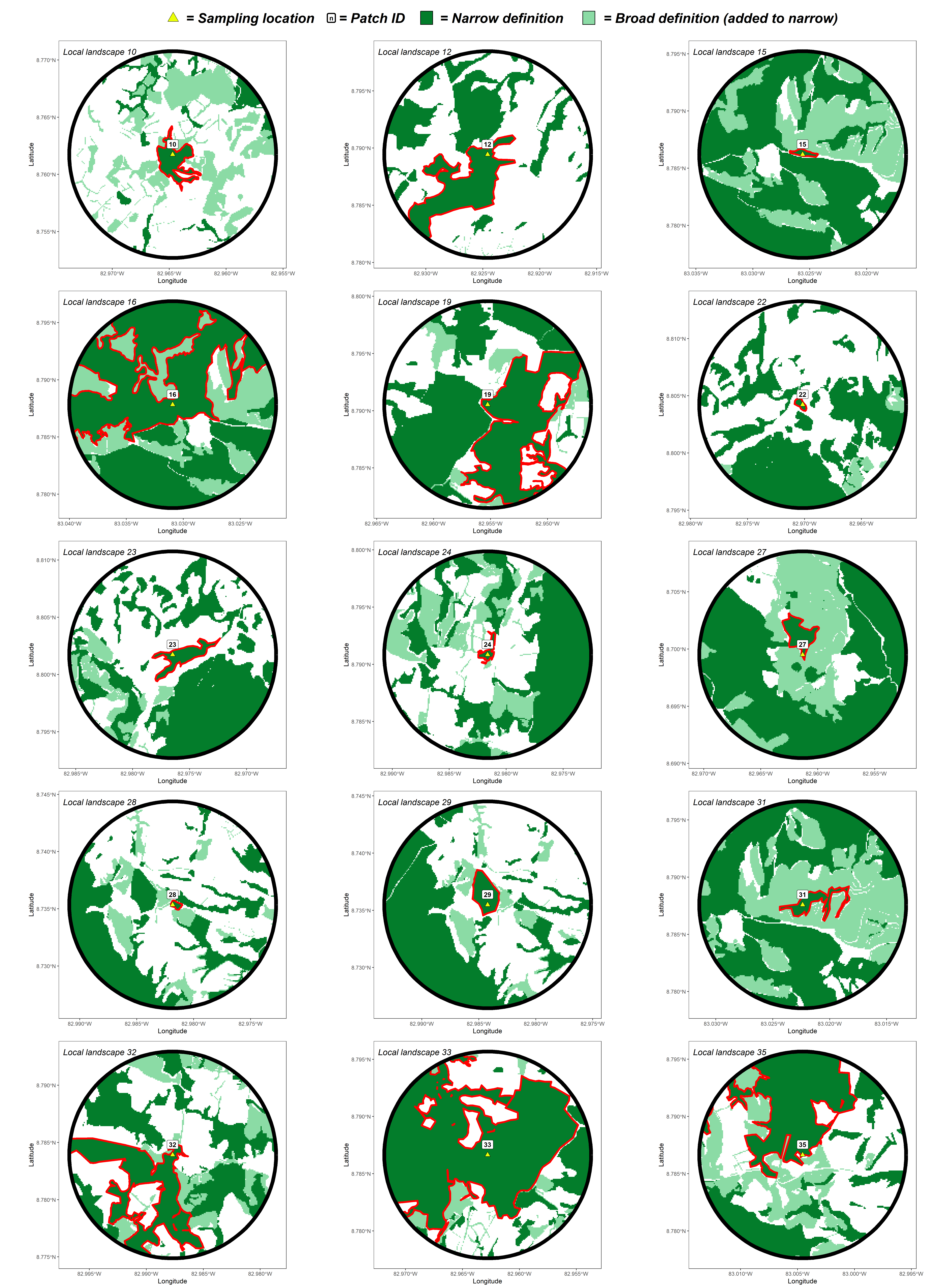


**Supplementary Figure S1:** Each local landscape (focal forest patch 10, 12, 15, 16, 19, 22, 23, 24, 27, 28, 29, 31, 32, 33, and 35) that surrounded each sampling site, defined as the area within a 1 km radius of the median GPS coordinate of the sampled maternal plants. Yellow triangles show the sampling sites, labeled with the ID of each focal forest patch. Dark green colors show pixels defined as hummingbird habitat by the ‘narrow’ definition (mature forest). Light green colors show pixels included as hummingbird habitat by the ‘broad’ definition (mature forest, regenerating forest, and narrow forest elements). The red line delimits the patch in which the samples were collected for each local landscape.


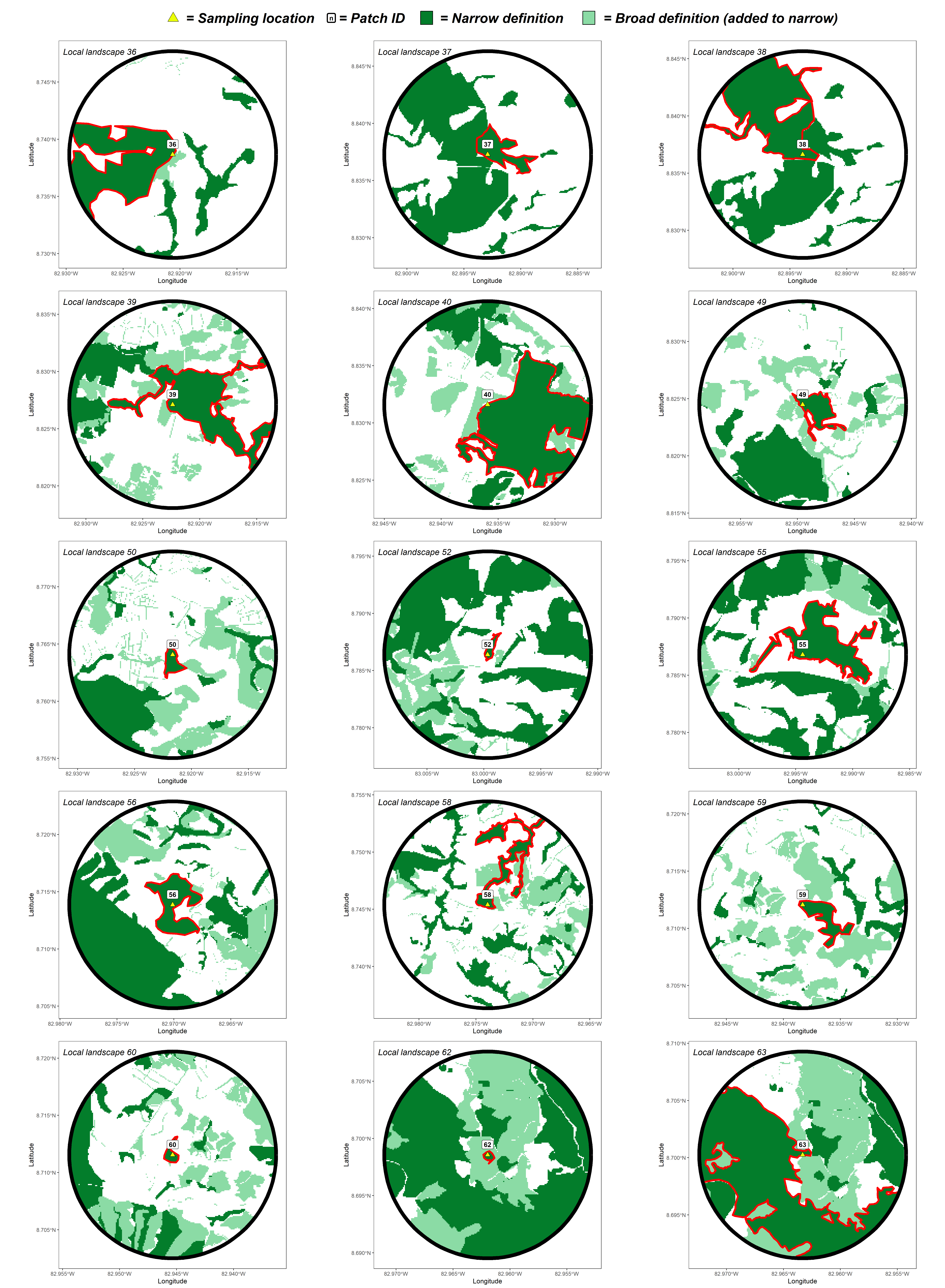


**Supplementary Figure S2:** Each local landscape (focal forest patch 36, 37, 38, 39, 40, 49, 50, 52, 55, 56, 58, 59, 60, 62, and 63) that surrounded each sampling site, defined as the area within a 1 km radius of the median GPS coordinate of the sampled maternal plants. Yellow triangles show the sampling sites, labeled with the ID of each focal forest patch. Dark green colors show pixels defined as hummingbird habitat by the ‘narrow’ definition (mature forest). Light green colors show pixels included as hummingbird habitat by the ‘broad’ definition (mature forest, regenerating forest, and narrow forest elements). The red line delimits the patch in which the samples were collected for each local landscape.

**Supplementary Methods and Results: Landscape Connectivity Models with Multiple Predictors – Correlations and Simulations**

We observed an important, positive correlation between the intra-patch and local landscape connectivity metrics (correlation coefficient ranged from 0.3 to 0.8). To determine if this correlation is a characteristic of the study area or a consequence of the behaviour of the landscape connectivity metrics that we conceptualized, we used the landscapeR package (Thomas *et al.*, 2020) to generate random local landscapes that included the observed area (ha) of the focal patches (0.6 ha to 200 ha) and the amount of forest cover within a 1 km radius (7.8% to 74.4%). We generated ten local landscapes for each combination of parameters and estimated all the landscape connectivity metrics described in Table 1. Then, we calculated the two most complex metrics: distance-weighted with both the gap-crossing threshold and probabilistic gap-crossing approaches (see Figure 1). We calculated these two metrics for both the whole local landscape (including the focal patch) and for only the non-focal patches in the local landscape. This in order to determine whether the influence of the focal patch could be removed from the local landscape connectivity metrics, enabling us to develop models with both an intra-patch and an extra-patch component.

From the simulated local landscapes, we found that the area of the focal patches and the local landscape connectivity metrics $S_{i}$ had a correlation coefficient of *r =* 0.74 for the gap-crossing threshold approach and *r =* 0.79 for the probabilistic gap-crossing approach. When removing the focal patch from the calculation of the $S_{i}$ metric, the correlation became negative (*r* = -0.34 and *r =* -0.31, respectively). This suggested that the $S_{i}$ metric that we conceptualized was limited by the available area outside of the focal patch, but within the 1 km local landscape. A plot of the area of the focal patches against the landscape connectivity metrics $S_{i}$ revealed a saturating relationship, where the rate of increase in landscape connectivity decreased as the area of the focal patch increased (Figure S3). Due to this correlation between focal patch area and local landscape connectivity metrics, we opted not to proceed with landscape connectivity models that included both intra-patch metrics and local landscape metrics, as the strong correlation between the two would have confounded the models.

**References**

**Thomas A, Masante D, Jackson B, Cosby B, Emmett B, Jones L**. **2020**. Fragmentation and thresholds in hydrological flow-based ecosystem services. *Ecological Applications* **30**: e02046.


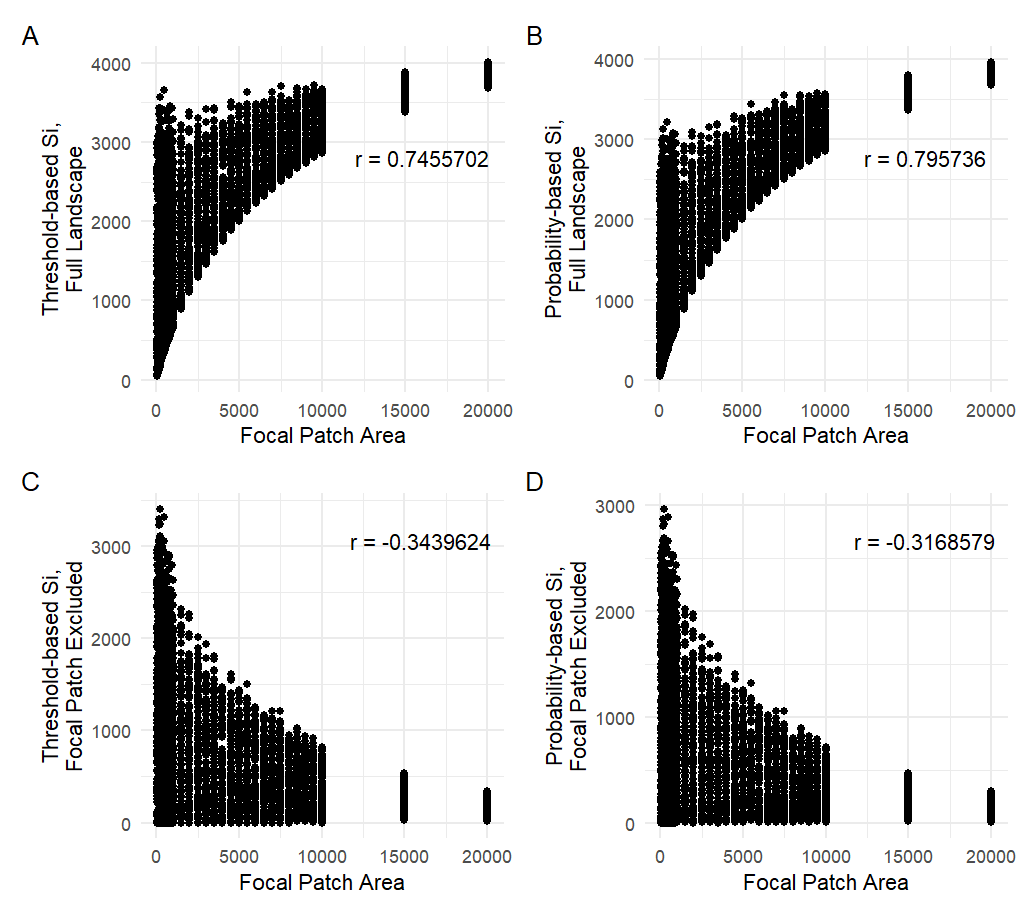


**Supplementary Figure S3:** Graphs depicting the relationship between focal patch area and distance-weighted $S_{i}$ metrics. (A and B) Full local landscape connectivity metrics (including the focal patch) show a strong positive correlation with focal patch area (Pearson’s correlation coefficient). (C and D) Local landscape connectivity metrics calculated only for non-focal patches show a moderate, but still substantial, negative correlation with focal patch area.


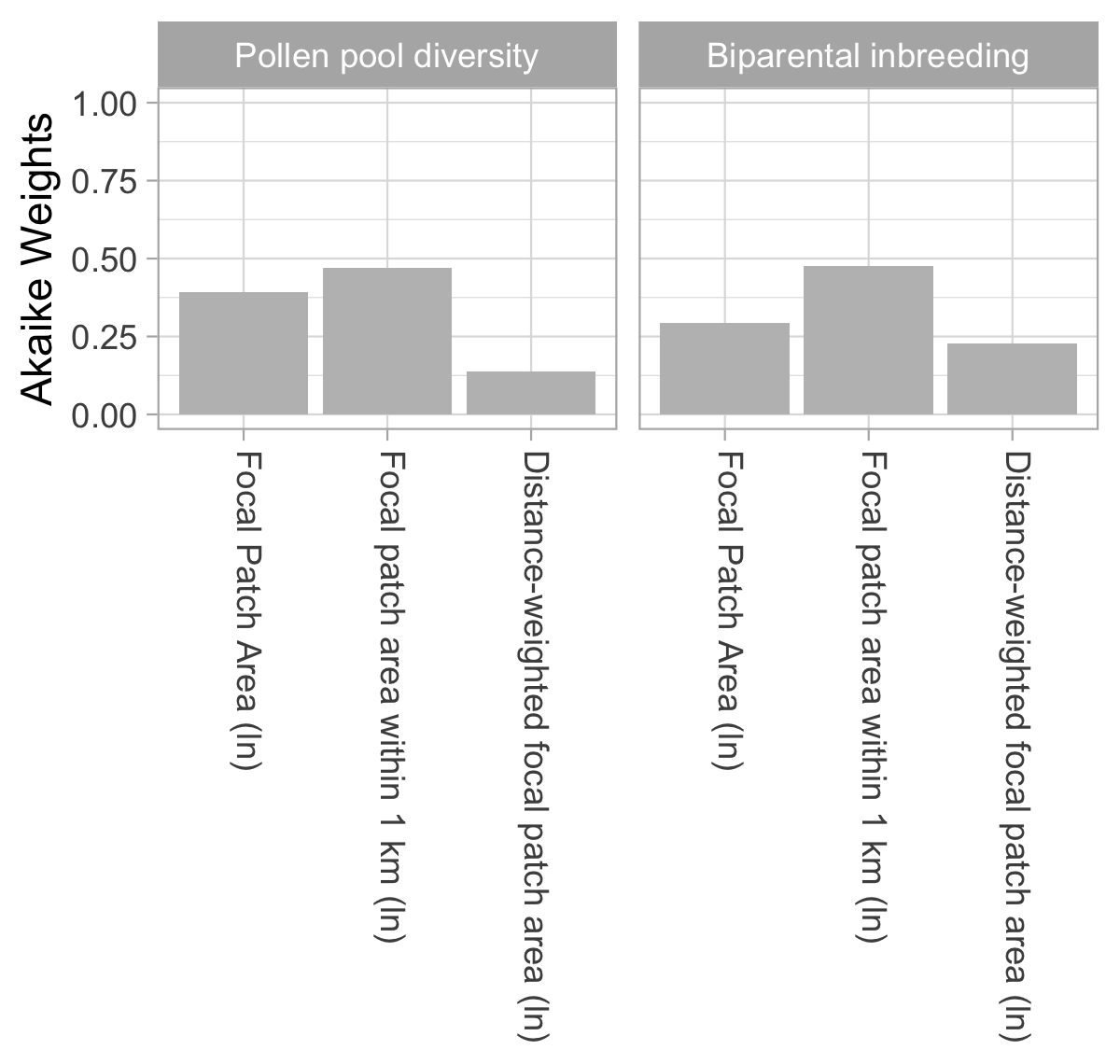


**Supplementary Figure S4:** Relative importance of intra-patch connectivity metrics for those patches with hummingbird capture data (n = 13). Each bar shows the Akaike weight (Table 2) of a regression model with an intra-patch connectivity metric as a single predictor, separately for each response variable (facets). Within each facet, weights sum to 1.


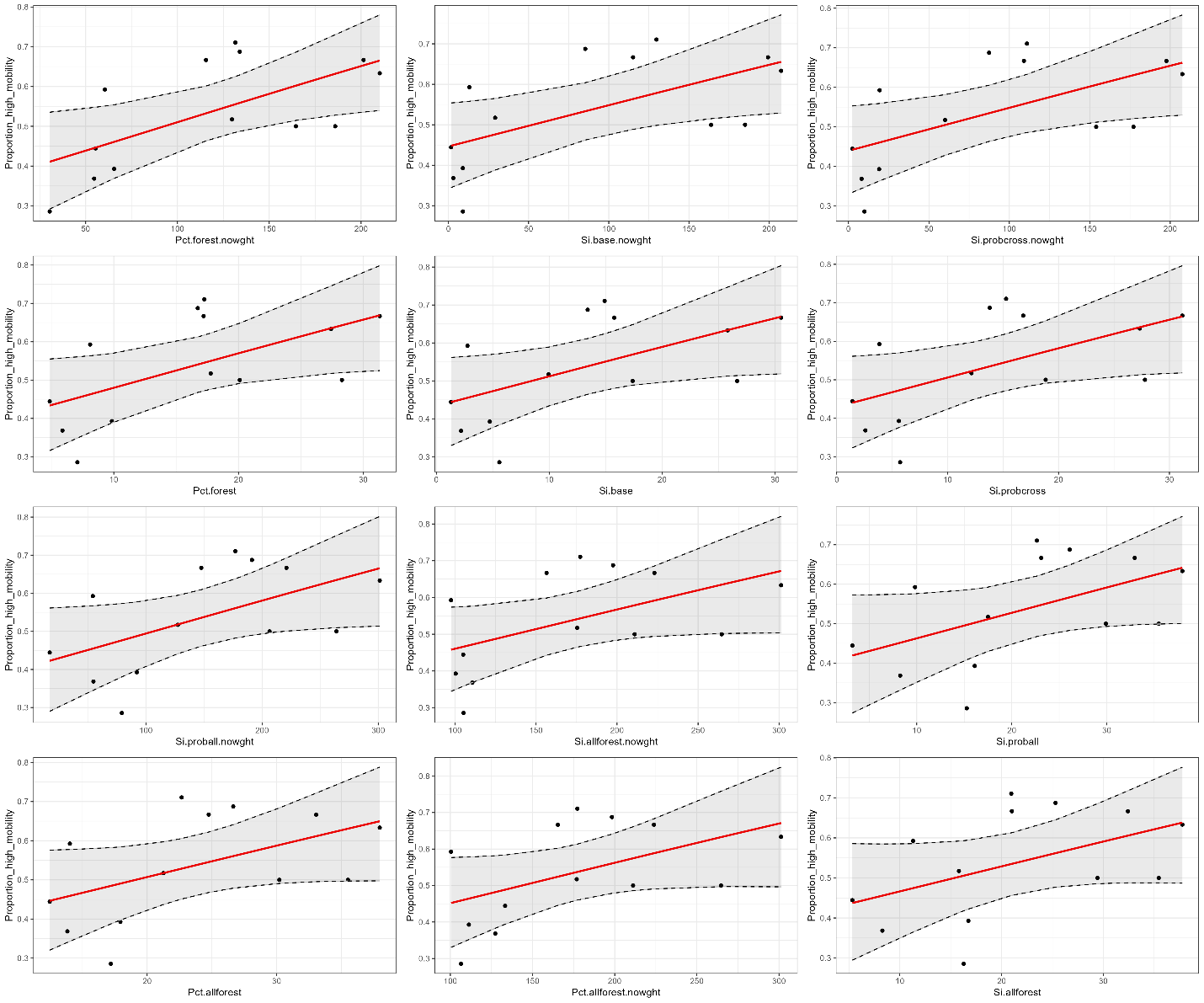


**Supplementary Figure S5:** Relationships between local landscape connectivity metrics and the proportion of high-mobility hummingbirds. Each panel includes a separate local landscape connectivity metric. The upper left panel shows the best model as evaluated by AICc values (also shown in Figure 3A), followed by models with increasing $\Delta{AIC}_{C}$ going from upper left to lower right panel. The solid red lines represent predicted values generated from the respective fitted models across the observed range of each predictor variable, and the dashed lines represent the 95% confidence intervals of the predicted values. The points correspond to the observed empirical data, which are based on the subset of 13 focal patches with hummingbird capture data.


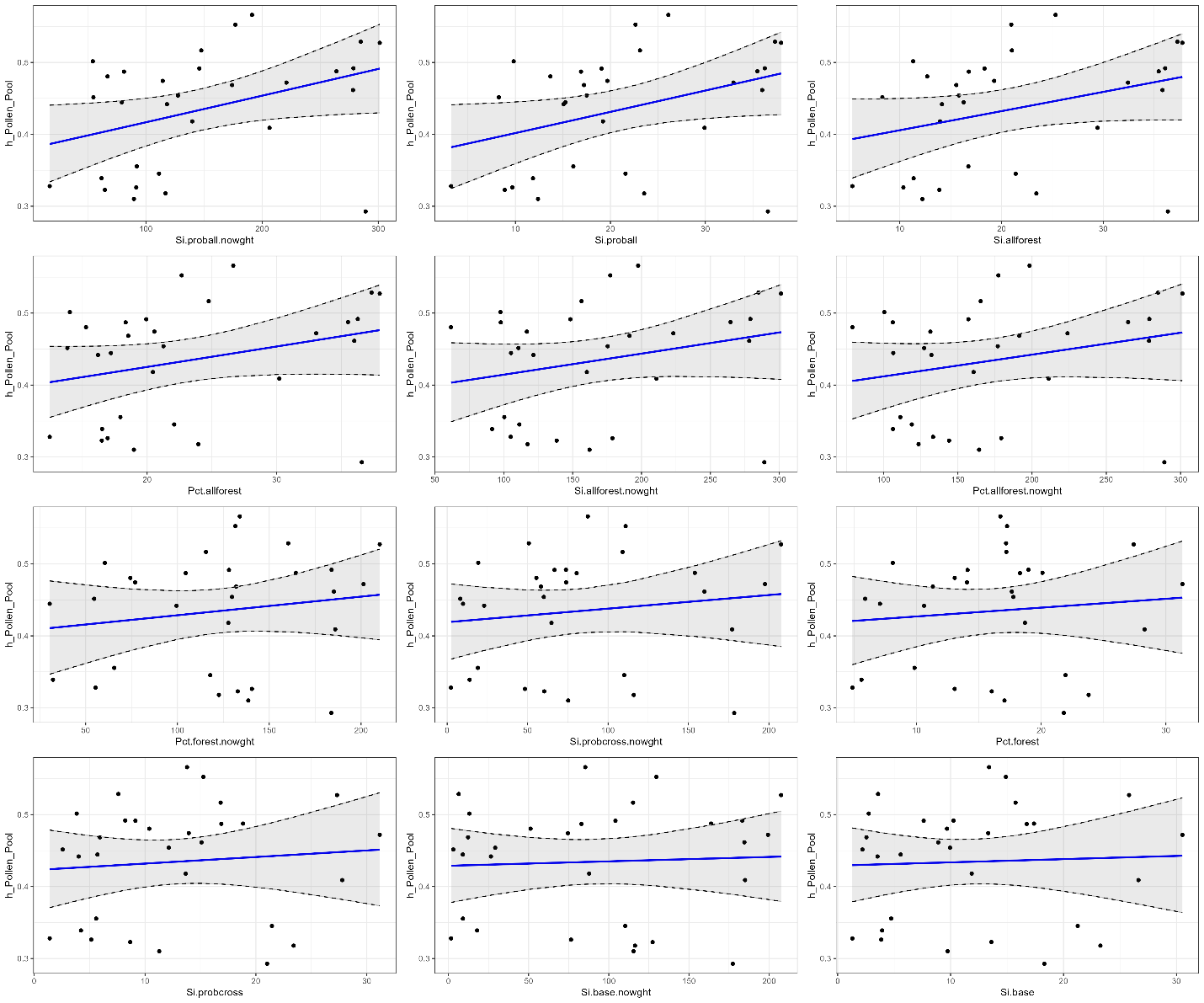


**Supplementary Figure S6:** Relationships between local landscape connectivity metrics and the haplotype diversity (h) of pollen pools. Each panel includes a separate local landscape connectivity metric. The upper left panel shows the best model as evaluated by AICc values (also shown in Fig. 3B), followed by models with increasing $\Delta{AIC}_{C}$ going from upper left to lower right panel. The solid blue lines represent predicted values generated from the respective fitted models across the observed range of each predictor variable, and the dashed lines represent the 95% confidence intervals of the predicted values. The points correspond to the observed empirical data, which are based on the complete set of 30 focal patches.


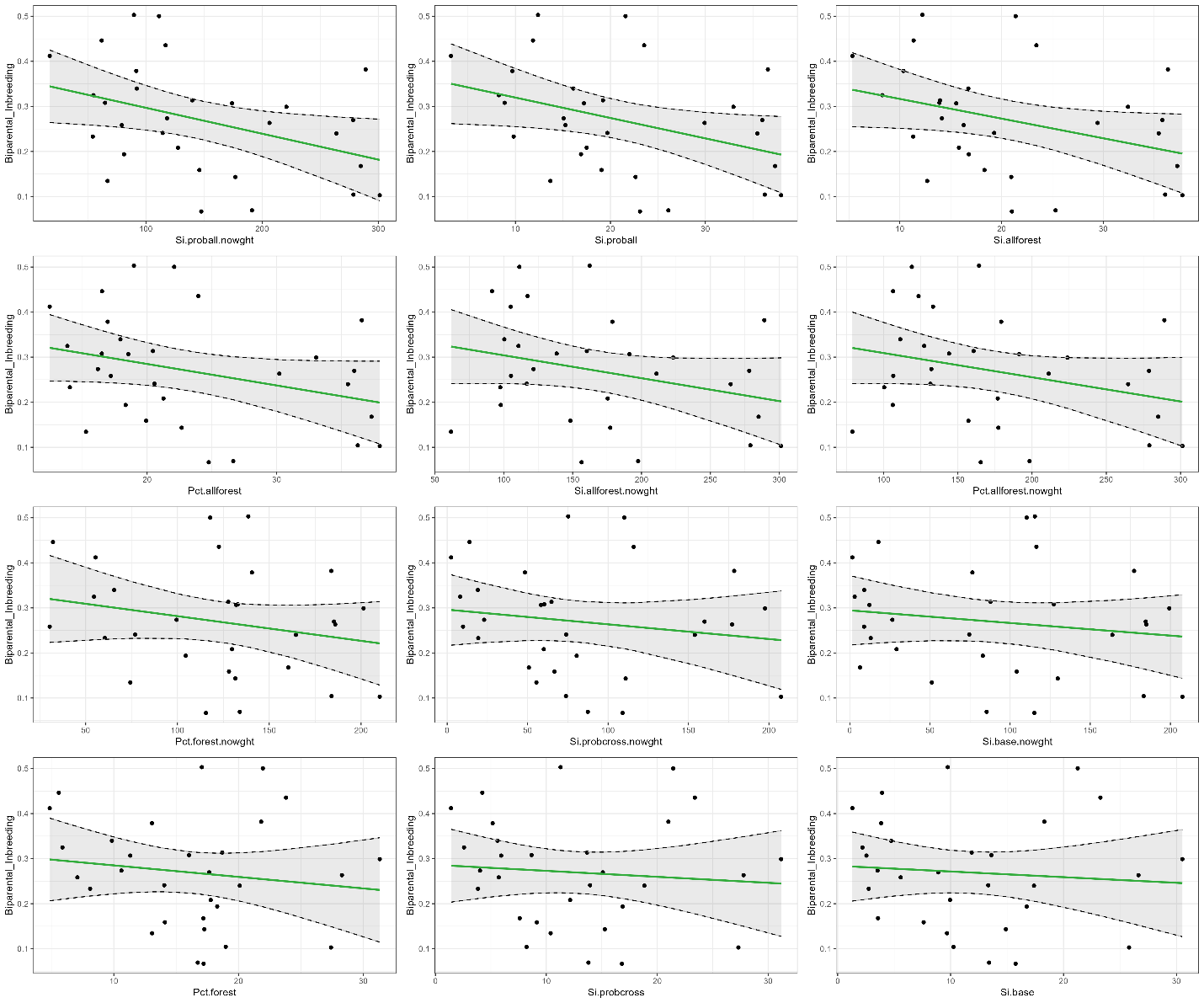


**Supplementary Figure S7:** Relationships between local landscape connectivity metrics and biparental inbreeding (t_m_ - t_s_). Each panel includes a separate local landscape connectivity metric. The upper left panel shows the best model as evaluated by AICc values (also shown in Fig. 3C), followed by models with increasing $\Delta{AIC}_{C}$ going from upper left to lower right panel. The solid green lines represent predicted values generated from the respective fitted models across the observed range of each predictor variable, and the dashed lines represent the 95% confidence intervals of the predicted values. The points correspond to the observed empirical data, which are based on the complete set of 30 focal patches.


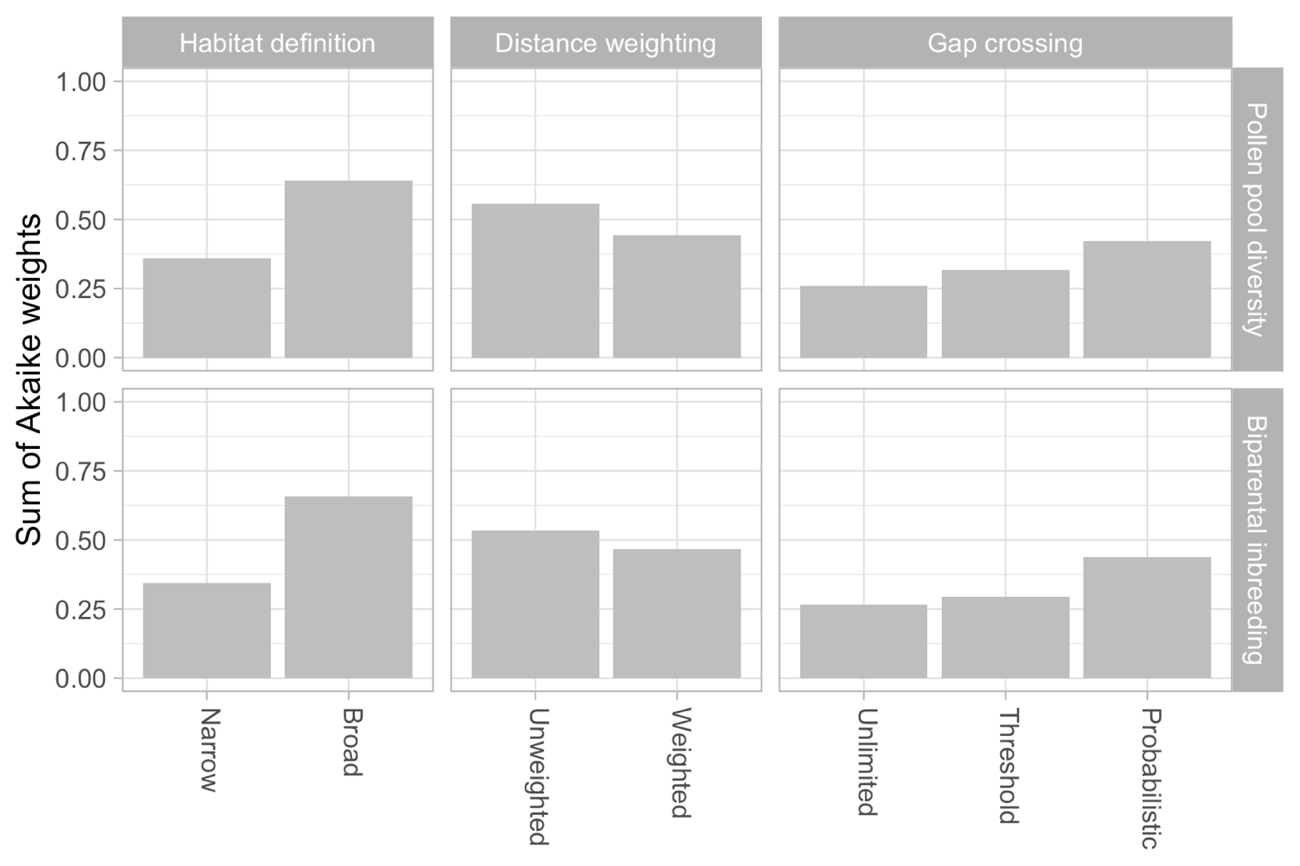


**Supplementary Figure S8:** Summed Akaike weights for local landscape connectivity metrics, for those patches with hummingbird capture data (n = 13). The facets represent the three factors that modified different aspects of functional connectivity (columns: habitat definition, distance-weighting, and gap-crossing) and the three response variables (rows). Each bar shows the sum of the Akaike weights of all local landscape connectivity metrics that included the respective factor level (i.e., sum over six models for two-level factors or sum over four models for three-level factors). Within each facet, bar heights sum to 1, and the height of a bar indicates the empirical support for a factor level.

**Supplementary Table S1:** Model comparison for intra-patch connectivity metrics calculated for the n = 13 patches with hummingbird capture data, separately for each genetic response variable. For each metric, summary results of a simple linear regression model are listed. Slope coefficients are beta coefficients, shown with their standard error,$R^{2}$ refers to the unadjusted coefficient of determination, AICc indicates the Akaike information criterion with sample size correction, $\Delta AICc$ refers to the difference between a model’s AICc value and the lowest AICc value for the same response variable, and model weight w expresses the relative support for each of the three models for the same response variable. The best-supported model for each response variable is presented in bold.

| **Response variable** | **Distance limitation** | **Metric** | **Slope ± SE** | ***R^2^*** | ***AICc*** | $\boldsymbol{\Delta}\boldsymbol{AICc}$ | ***w*** |
| --- | --- | --- | --- | --- | --- | --- | --- |
| Pollen pool diversity  (haplotype diversity *h*) | Unlimited | Focal patch area (ln) | 0.637 ± 0.232 | 0.406 | 37.76 | 0.35 | 0.394 |
|  | Threshold 1 km | Focal patch area within 1 km radius (ln) | **0.649 ± 0.229** | **0.421** | **37.41** | **0.00** | **0.469** |
|  | Weighted | Distance-weighted focal patch area | 0.549 ± 0.252 | 0.301 | 39.86 | 2.46 | 0.137 |
| Biparental inbreeding  (*t_m_* - *t_s_*) | Unlimited | Focal patch area (ln) | -0.623 ± 0.236 | 0.389 | 38.12 | 0.96 | 0.295 |
|  | Threshold 1 km | Focal patch area within 1 km radius (ln) | **-0.657 ± 0.227** | **0.432** | **37.16** | **0.00** | **0.476** |
|  | Weighted | Distance-weighted focal patch area | -0.604 ± 0240 | 0.364 | 38.63 | 1.47 | 0.229 |

**Supplementary Table S2:** Model comparison for local landscape connectivity metrics calculated for the n = 13 patches with hummingbird capture data, separately for each genetic response variable. For each metric (row), summary results of a simple linear regression model are listed. The slope coefficients are beta coefficients, shown with their standard error (SE). The R^2^ refers to the unadjusted coefficient of determination and the AIC_C_ indicates the Akaike information criterion with sample size correction. The ΔAIC_C_, represents the difference between a model’s AIC_C_ value and the lowest AIC_C_ value for the same response variable. The model weights w represents the relative support for each of the twelve local landscape connectivity metrics for the same response variable. The best-supported model for each response variable is shown in bold.

| **Response variable** | **Habitat definition** | **Distance weighting** | **Gap-crossing** | ***Slope ±* *SE*** | ***R^2^*** | ***AICc*** | $\boldsymbol{\Delta}\boldsymbol{AICc}$ | ***w*** |
| --- | --- | --- | --- | --- | --- | --- | --- | --- |
| Pollen pool diversity (haplotype diversity *h*) | Narrow | Unweighted | Unrestricted | 0.404 ± 0.276 | 0.163 | 42.21 | 2.06 | 0.063 |
|  |  |  | Threshold | 0.435 ± 0.271 | 0.189 | 41.79 | 1.65 | 0.077 |
|  |  |  | Probabilistic | 0.423 ± 0.273 | 0.179 | 41.96 | 1.82 | 0.071 |
|  |  | Weighted | Unrestricted | 0.354 ± 0.282 | 0.126 | 42.77 | 2.63 | 0.047 |
|  |  |  | Threshold | 0.363 ± 0.281 | 0.132 | 42.68 | 2.54 | 0.05 |
|  |  |  | Probabilistic | 0.365 ± 0.281 | 0.133 | 42.66 | 2.52 | 0.05 |
|  | Broad | Unweighted | Unrestricted | 0.417 ± 0.274 | 0.174 | 42.04 | 1.90 | 0.068 |
|  |  |  | Threshold | 0.473 ± 0.266 | 0.224 | 41.22 | 1.08 | 0.103 |
|  |  |  | **Probabilistic** | **0.535 ± 0.255** | **0.286** | **40.14** | **0.00** | **0.176** |
|  |  | Weighted | Unrestricted | 0.441 ± 0.271 | 0.195 | 41.7 | 1.56 | 0.081 |
|  |  |  | Threshold | 0.454 ± 0.269 | 0.206 | 41.52 | 1.38 | 0.088 |
|  |  |  | Probabilistic | 0.498 ± 0.262 | 0.248 | 40.82 | 0.68 | 0.126 |
| Biparental inbreeding (*t_m_* - *t_s_*) | Narrow | Unweighted | Unrestricted | -0.417 ± 0.274 | 0.174 | 42.03 | 2.14 | 0.062 |
|  |  |  | Threshold | -0.425 ± 0.273 | 0.181 | 41.92 | 2.04 | 0.065 |
|  |  |  | Probabilistic | -0.428 ± 0.273 | 0.183 | 41.89 | 2.01 | 0.066 |
|  |  | Weighted | Unrestricted | -0.380 ± 0.279 | 0.145 | 42.49 | 2.60 | 0.049 |
|  |  |  | Threshold | -0.379 ± 0.279 | 0.144 | 42.50 | 2.61 | 0.049 |
|  |  |  | Probabilistic | -0.389 ± 0.278 | 0.151 | 42.38 | 2.50 | 0.052 |
|  | Broad | Unweighted | Unrestricted | -0.427 ± 0.273 | 0.183 | 41.90 | 2.01 | 0.066 |
|  |  |  | Threshold | -0.475 ± 0.265 | 0.225 | 41.20 | 1.31 | 0.094 |
|  |  |  | **Probabilistic** | **-0.547 ± 0.252** | **0.300** | **39.89** | **0.00** | **0.180** |
|  |  | Weighted | Unrestricted | -0.469 ± 0.266 | 0.220 | 41.29 | 1.40 | 0.089 |
|  |  |  | Threshold | -0.466 ± 0.267 | 0.217 | 41.33 | 1.45 | 0.088 |
|  |  |  | Probabilistic | -0.521 ± 0.257 | 0.271 | 40.40 | 0.51 | 0.140 |
